# mRNA association by aminoacyl tRNA synthetase occurs at a putative anticodon mimic and autoregulates translation in response to tRNA levels

**DOI:** 10.1371/journal.pbio.3000274

**Published:** 2019-05-17

**Authors:** Ofri Levi, Yoav Arava

**Affiliations:** Faculty of Biology, Technion, Israel Institute of Technology, Haifa, Israel; Case Western Reserve University, UNITED STATES

## Abstract

Aminoacyl-tRNA synthetases (aaRSs) are well studied for their role in binding and charging tRNAs with cognate amino acids. Recent RNA interactome studies had suggested that these enzymes can also bind polyadenylated RNAs. Here, we explored the mRNA repertoire bound by several yeast aaRSs. RNA immunoprecipitation (RIP) followed by deep sequencing revealed unique sets of mRNAs bound by each aaRS. Interestingly, for every tested aaRSs, a preferential association with its own mRNA was observed, suggesting an autoregulatory process. Self-association of histidyl-tRNA synthetase (HisRS) was found to be mediated primarily through binding to a region predicted to fold into a tRNA^His^ anticodon-like structure. Introducing point mutations that are expected to disassemble this putative anticodon mimic alleviated self-association, concomitant with increased synthesis of the protein. Finally, we found that increased cellular levels of uncharged tRNA^His^ lead to reduced self-association and increased HisRS translation, in a manner that depends on the anticodon-like element. Together, these results reveal a novel post-transcriptional autoregulatory mechanism that exploits binding mimicry to control mRNA translation according to tRNA demands.

## Abstract 2

Better known for their enzymatic role in charging tRNAs with their cognate amino acids, this study shows that tRNA synthetases also bind mRNAs, regulating translation in order to balance the production of a tRNA synthetase with the level of its cognate tRNA.

## Introduction

RNA-binding proteins (RBPs) encompass a significant fraction of an organism proteome and are implicated in many cellular processes [1]. The group of RBPs that bind mRNA is likely to be most critical for expression regulation. In the course of an mRNA’s maturation, different RBPs bind the transcript and mediate its nuclear processing, its export out of the nucleus, cellular localization, translation, and degradation [2,3]. Therefore, identification and characterization of novel mRNA-binding protein (mRBPs) is a central goal in molecular biology.

Recent high-throughput studies applied an unbiased approach whereby polyadenylated RNAs and their associated proteins were isolated through an oligo dT column and proteins were identified by mass spectrometry [4,5]. These RNA interactome studies identified many novel mRBPs from various organisms and cell types [6–13]. Interestingly, many of these novel mRBPs are enzymes with well-established metabolic roles of which mRNA regulation appeared as an additional function [14,15].

A recurring family of enzymes that was observed among these studies is the family of cytosolic aminoacyl-tRNA synthetases (aaRSs). aaRSs recognize their cognate tRNA and amino acid and perform the charging reaction of the amino acid at the 3′ end of the tRNA with the expanse of an ATP [16]. Most cells have 20 different cytosolic aaRSs, each one responsible to ligate an amino acid to its cognate tRNAs [17]. Cells also encode aaRSs that are localized in mitochondria and charge tRNAs that are transcribed by the mitochondrial genome. Previous studies revealed noncatalytic functions for some aaRSs, including transcription regulation, cellular signaling, and mitochondrial splicing (reviewed by [16,18]). Three aaRSs, from diverse species, were documented to bind mRNA and exert a post-transcriptional role: the mammalian glutamyl-prolyl-tRNA synthetase (GluProRS) is part of a multiprotein complex that regulates mRNA expression through binding to the 3′ UTR of mRNAs [19], and its mechanism of action and activation pathway are well studied [20]. The *Escherichia coli* threonyl-tRNA synthetase (ThrRS) can bind its own mRNA in a region upstream to the start codon (“operator” region) [21,22] and autoregulates its translation. *Saccharomyces cerevisiae* aspartyl-tRNA synthetase (AspRS) was also found to bind in vitro its own mRNA [23] and to regulate its nuclear export upon overexpression of tRNA^Asp^ [24].

The abundant detection of aaRS in interactome studies suggests that mRNA binding is far more common than the three cases mentioned above. For example, all 20 aaRSs were detected by mass spectrometry as bound to polyA RNA in at least one of the analyses done for *S*. *cerevisiae* (S1 Table), yet only AspRS was known to do so before. Furthermore, only a single mRNA target was characterized for each of these synthetases (*E*.*coli* ThrRS and *S*. *cerevisiae* AspRS bind their own mRNA, and GluProRS binds the ceruloplasmin mRNA [*Cp* mRNA]); therefore, the generality of mRNA association is not clear.

Herein applied an in vivo genomic approach to identify the mRNA targets of several *S*. *cerevisiae* aaRSs. RNA immunoprecipitation (RIP) followed by RNA-seq revealed that each aaRS binds a unique subset of mRNAs. Moreover, all tested aaRSs appeared to bind their own mRNA much better than most other mRNAs. Histidyl-tRNA synthetase (HisRS) had the most striking self-association and appeared to bind its own mRNA orders of magnitude higher than any other mRNA. Mapping the mRNA-binding site revealed a site with high similarity to the tRNA^His^ anticodon loop, and a set of mutations confirmed the importance of this anticodon mimic for binding. We further show that binding represses translation of HisRS, and this repression is alleviated upon increase in tRNA levels. These data present a novel autoregulatory loop that controls translation through protein binding to an anticodon mimic within the mRNA.

## Results

### aaRSs bind diverse sets of mRNAs

We analyzed data from three RNA interactome studies performed for *S*. *cerevisiae* [7,10,11] and found that all 20 cytosolic aaRSs were detected in at least one study and the vast majority in two or all three studies (S1 Table). We therefore aimed at exploring the repertoire of mRNAs bound by aaRSs. To this end, we utilized yeast strains expressing endogenously, C-terminally Tandem Affinity Purification (TAP)-tagged methionyl-tRNA synthetase (MetRS), glutamyl-tRNA synthetase (GluRS), valyl-tRNA synthetase (ValRS), and HisRS or green fluorescent protein (GFP)-tagged HisRS and glutamine-tRNA synthetase (GlnRS). These aaRSs appeared to be associated with mRNA in at least two of the yeast interactome studies (S1 Table). Cells were grown to midlogarithmic phase, cross-linked by formaldehyde, and subjected to RIP with anti-GFP conjugated beads (GFP-Trap) or proteinA beads (for TAP fusions). Protein samples from the initial extract (Input), flow-through of the beads (FT), last washing step (LW), and the bound material (Bound) were analyzed by polyacrylamide gel (PAGE) followed by western analysis (S1A Fig) and revealed high efficiency and specificity of the isolation protocol. This was also demonstrated for HisRS-GFP by silver staining (S1B Fig), whereby the only detected band in the Bound sample is of the HisRS-GFP protein.

**Fig 1:**
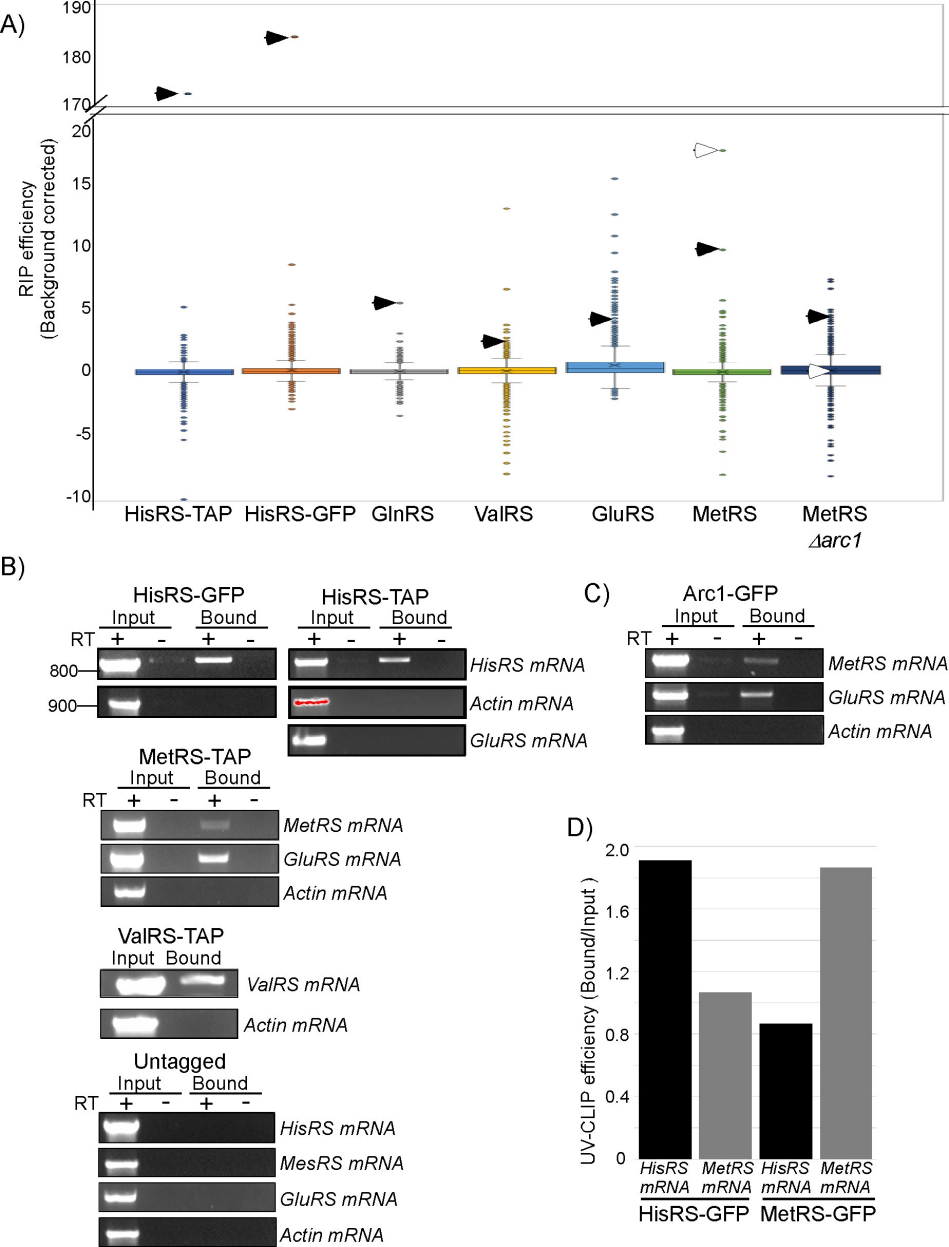
mRNA binding by aaRSs. A) Yeast strains expressing the indicated tagged aaRS (either TAP or GFP tagged) or an untagged control strain were subjected to RIP-seq analysis. The box plot presented the RIP efficiency (calculated as RPM in the Bound sample divided by RPM in the Input) corrected for background (i.e., subtracted by the RIP efficiencies obtained by the untagged strain RIP-seq) from a single experiment. Extreme values are presented by circles, and whiskers correspond to 1.5 IQR. Closed arrowheads point the position of the mRNA encoding the respective aaRS. Open arrowheads point the mRNA encoding GluRS. See S2 Table for complete data set. B) Semiquantitative RT-PCR confirmation of positive hits. RNA was extracted from either the Input sample or the Bound RNA and subjected to reverse transcription either with (+) or without (-) the RT enzyme. cDNA was subjected to PCR with primers specific to the indicated genes, and products were collected every five cycles and resolved on an agarose gel. Representative gel images from the same PCR cycle are presented. Data are representative of two independent biological repeats. Red shading for the Actin signal in the Input sample of HisRS-TAP indicates saturation. C) Semiquantitative RT-PCR for RIP analysis of Arc1-GFP strain. D) Quantitative RT-PCR for the indicated mRNAs, isolated by UV cross-linking, followed by IP from HisRS-GFP and MetRS-GFP strains. Results are presented as the ratio between Bound and Input RT-qPCR signals and are representative of two independent biological repeats. Raw data is presented in S6 Table. aaRS, aminoacyl-tRNA synthetase; GFP, green fluorescent protein; GluRS, glutamyl-tRNA synthetase; HisRS, histidyl-tRNA synthetase; IP, immunoprecipitation; IQR, InterQuartile Range; MetRS, methionyl-tRNA synthetase; RIP, RNA immunoprecipitation; RPM, reads per million; RT-PCR, reverse transcription PCR; TAP, Tandem Affinity Purification.

RNA samples were prepared from before the purification (Input), representing the total transcriptome of the strain, and from the Bound sample, representing the transcripts that are associated with each aaRS. As a control for nonspecific association with the beads, we performed similar isolation procedures (TAP and GFP-Trap) to an untagged strain (BY4741). RNA samples from the Input and Bound fractions from all tagged and untagged strains were subjected to RNA-seq, and signals for about 5,000 different mRNAs were obtained in each (S2 Table). mRNAs that were efficiently bound by each aaRS were determined by calculating the ratio between Bound and Input reads for each mRNA (“RIP efficiency”) (S2 Table). The division by the Input reads accounts for differences in abundance and length between mRNAs. Analysis of the top ranked mRNAs in each RIP-seq revealed that many target mRNAs are shared among several aaRS (S2 Fig). However, the vast majority of these also appeared to be highly ranked in the RIP-seq analysis of the corresponding untagged strain (S2A Fig and S2B Fig); therefore, their significance is unclear. To account for such non-aaRS–mediated interaction with the beads, we subtracted for each gene its observed RIP efficiency in the untagged strain. This resulted in a background-corrected RIP efficiency value for each mRNA (Fig 1A and S2 Table).

GluRS appeared to have the largest number of targets (213 mRNAs, after excluding those that appeared also in the untagged strain RIP-seq). Ninety-nine of these were bound exclusively by GluRS (within 1.5 InterQuartile Range [IQR] above the average) (S3 Table). Gene Ontology (GO) term analysis for these revealed that GluRS preferentially binds mRNAs that encode proteins with Transcription Regulator activity (S4 Table). Interestingly, the 62 shared targets of GluRS and HisRS were also enriched for this family (S2A Fig and S4 Table). This suggests that GluRS acts as a post-transcriptional regulator of transcription factors and may work solo on some targets and together with HisRS on other targets.

GluRS also shares 11 target mRNAs with MetRS, including the mRNAs encoding GluRS and MetRS themselves (S3 Table) (see below). Several other aaRSs shared a large number of mRNA targets, including MetRS and ValRS (24 targets), MetRS and HisRS (12 targets), and HisRS and GlnRS (26 targets) (S2 Fig and S3 Table). The group bound by HisRS and GlnRS included 16 mRNAs encoding proteins with ion-binding activity (GO: 00431670), including the mRNAs encoding AspRS and GlnRS and the serine/threonine kinases VPS15 and PKH2 (S3 Table). However, the processes that these 16 ion binders are involved in appear very diverse and with no obvious common features. Overall, their significance necessitates further investigation.

mRNAs bound exclusively by MetRS appeared to be highly enriched for those encoding proteins that bind ATP (32 of the 115 mRNAs) (S4 Table). MetRS was shown previously to have a role in activation of transcription of ATP synthesis genes upon yeast diauxic shift [25]. Our data suggest an additional role in post-transcriptional regulation of mRNAs encoding proteins that utilize ATP for their activity. MetRS might therefore be a coordinator of ATP synthesis by ATP synthase and ATP utilization by ATPases.

### aaRSs preferentially bind their own mRNA

A common feature that appeared for each aaRSs that we tested was a strong association with its own mRNA (“self-association”) (Fig 1A). HisRS (by either TAP or GFP protocols) and GlnRS were ranked first among approximately 5,000 mRNAs that appeared as bound by each protein, MetRS mRNA was ranked second of 5,089 mRNAs that appeared as bound by MetRS protein, ValRS 16 of 5,104, and GluRS mRNA was ranked 76 of 5,124 (Fig 1A and S2 Table). Self-association may imply an autogenous post-transcriptional regulatory process. The fact that self-association was observed for every aaRS that we tested suggests that this process is common to all *S*. *cerevisiae* aaRSs.

To validate these sequencing results by an alternative approach, we performed semiquantitative reverse transcription PCR (RT-PCR) to RNA samples from the Input or Bound samples (Fig 1B). cDNA from the indicated preparations was subjected to PCR with specific primers, and aliquots from the same PCR cycle were resolved in agarose gel. Clear signal for HisRS is apparent in the Bound sample from either its GFP or TAP samples. This is not due to a contaminating DNA since no signal is detected in the control reaction without the RT enzyme. Importantly, while Actin signal appears much higher in the Input sample, no signal is detected in any of the Bound samples. Similarly, MetRS and ValRS mRNAs are detected in their respective RIP. Furthermore, the mRNA for GluRS is detected as highly associated with MetRS, even higher than MetRS mRNA. This is consistent with the RIP-seq data of MetRS-TAP, in which GluRS mRNA is ranked higher (first) than MetRS mRNA (second) (Fig 1A and S2 Table). Overall, these results are consistent with the relative associations of aaRSs that were observed by the RIP-seq analysis and confirm that aaRSs are associated with their own mRNA.

GluRS and MetRS are known to charge their cognate tRNAs while in a trimeric complex with Arc1 (Arc1:MetRS:GluRS [AME] complex) [26,27]. The coisolation of their mRNAs suggest that also their mRNA binding occurs while in a complex. We thus performed RIP followed by semiquantitative RT-PCR for GFP-tagged Arc1 protein. Clear signal of mRNAs encoding both complex partners, MetRS and GluRS, was detected (Fig 1C). Recently, Shiber A. and colleagues [28] had shown that assembly of the AME complex occurs cotranslationally, whereby MetRS interacts with the nascent chain of GluRS as it emerges from the ribosome and vice versa for GluRS. This process generates indirect interactions between MetRS and the translated mRNAs of GluRS and thus may explain our RIP results. Intriguingly, however, neither GluRS nor MetRS were found to associate with their own nascent chain [28]. This may suggest that the self-association that we observe for GluRS or MetRS are indirect ones, in which isolation of MetRS-TAP leads to isolation of the entire AME complex, thus MetRS mRNA is isolated due to its association with GluRS protein. To test this possibility, we deleted Arc1 from MetRS-TAP strain (thus disassembling the trimeric complex [26,27]) and subjected it to RIP-seq analysis, as above. As can be seen in Fig 1A, the self mRNA was highly ranked even upon complex disassembly, while GluRS mRNA was significantly downgraded (Fig 1A). Thus, self-association is independent of the AME complex while partner association depends on complex assembly. These data therefore reveal at least two interaction modes for MetRS: a cotranslational-dependent one with GluRS mRNA and a cotranslational- and complex-independent one with self mRNA.

To obtain insights to whether self-association can be also observed by other cross-linking approaches, we applied UV cross-linking (which was proposed to induce direct interactions between RNA and proteins [29]) to HisRS-TAP cells. HisRS-TAP was subjected to varying RNaseI treatments and then immunoprecipitated (S3 Fig). HisRS–cross-linked RNAs were labeled at the 5' end by ^32^P and resolved in a gel. HisRS proteins of increased sizes are apparent due to their associated RNA fragments (S3B Fig). Importantly, when bound RNA was extracted from the protein and resolved on a gel, species larger than 100 nts were clearly detected (S3C Fig). Thus, HisRS binds RNA species that are longer than tRNAs. Quantitative RT-PCR (RT-qPCR) to target specific mRNAs that are associated with HisRS yielded positive signals for HisRS mRNA, much higher than a control mRNA (MetRS mRNA) (Fig 1D). Similar UV cross-linking–IP experiment with MetRS-GFP resulted the same, i.e., high signal for self mRNA and lower to the control, HisRS mRNA (Fig 1D). These results indicate that association, at least for these two examples, is likely through direct interaction of the synthetase with its mRNA.

### HisRS binds mRNA through its anticodon-binding domain

**Fig 2:**
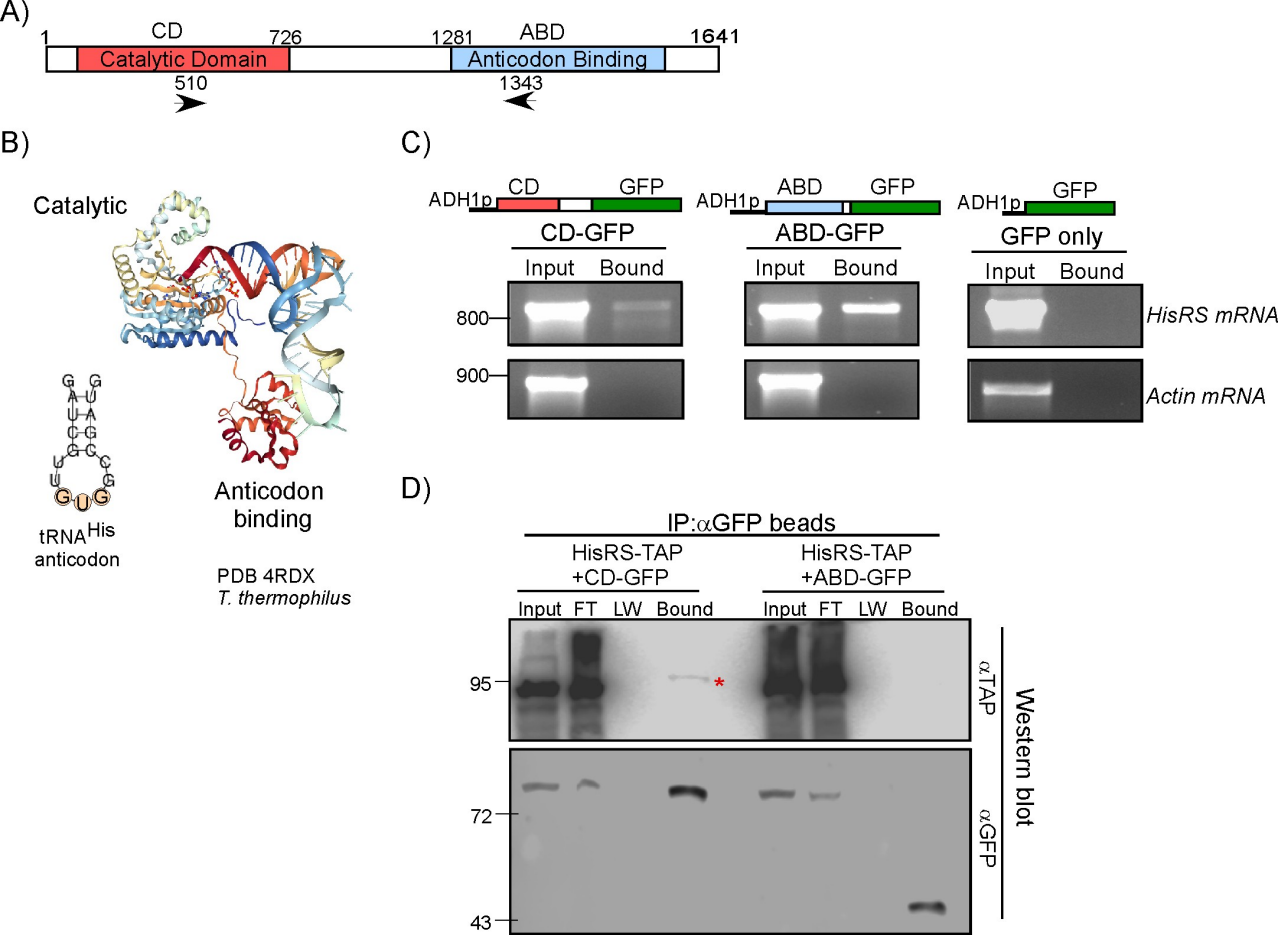
The ABD is the primary mRNA-binding domain. A) Protein domain structure of HisRS. Arrowheads indicate the positions of primers used for RT-PCR in panel C. B) Crystal structure of the *T*. *thermophilus* HisRS with its tRNA (PDB 4RDX) and the predicted structure of the tRNA^His^ anticodon arm. C) The CD and ABD were cloned upstream to GFP under the control of ADH1 promoter and introduced into BY4741 cells. Strains were subjected to GFP-Trap followed by semiquantitative RT-PCR to the Input and the Bound samples. Primers recognizing the endogenous HisRS mRNA (indicated as arrowheads in A) or Actin control mRNA were used. Images of agarose gel of PCR products from the same amplification cycle are presented. Numbers indicate the migration of a DNA size marker. D) CD-GFP or ABD-GFP plasmids were introduced into cells expressing endogenously TAP-tagged HisRS. Proteins were immunoprecipitated by GFP-Trap beads, and protein samples from the total cellular lysate (Input), unbound FT, LW step, and the eluted fraction (Bound) were resolved on PAGE, followed by western analysis with anti-TAP antibodies (top panel) or anti-GFP control for similar IP efficiency (bottom panel). Migration positions of protein size markers are indicated to the left. Red asterisk indicates HisRS-TAP that is copurified only with the CD region. ABD, anticodon-binding domain; ADH1, alcohol dehydrogenase 1; CD, catalytic domain; FT, flow-through; GFP, green fluorescent protein; HisRS, histidyl-tRNA synthetase; IP, immunoprecipitation; LW, last washing; PDB, Protein Data Bank; RT-PCR, reverse transcription PCR; TAP, Tandem Affinity Purification.

The self-association of HisRS appears to be in orders of magnitude higher compared to other cellular mRNAs (Fig 1A). This was apparent in both preparations from either the TAP- or GFP-tagged strains. The TAP and GFP-Trap protocols have several important differences, including the use of different antibodies, different beads, and a different elution method. Thus, self-association is independent of the purification protocol. We thus wished to determine the domain through which HisRS binds its mRNA. HisRS contains two domains that are important for proper charging of its tRNA: the catalytic domain (CD), which recognizes the acceptor stem and performs the charging reaction, and the anticodon-binding domain (ABD), which interacts with the anticodon loop (Fig 2A). Crystal structure of HisRS from *Thermus thermophilus* revealed that these are the primary contacts with tRNA (Fig 2B) [30]. To determine which of these domains is important for mRNA recognition, we fused each of them to GFP under the control of an alcohol dehydrogenase 1(ADH1) promoter. Plasmids were introduced into cells that also express an endogenous untagged HisRS transcript, and strains were subjected to GFP-Trap mRNA isolation. Samples were analyzed by semiquantitative PCR with primers that recognize only the endogenous transcript (Fig 2C). The results show an approximate 10-fold higher association of the mRNA with the ABD-GFP protein, indicating that mRNA recognition is mainly through the ABD. Notably, due to the primers design, the RT-PCR signals result only from the endogenous transcript, which is not GFP tagged. This demonstrates that HisRS protein interacts with mRNA in *trans*.

HisRS is known to efficiently dimerize, which raises the possibility of cotranslational association between the fully synthesized protein and a nascent dimerization domain. This may lead to a cotranslational dependent isolation of mRNA, as described for GluRS mRNA with MetRS-GFP. To explore this possibility, we introduced either CD-GFP or ABD-GFP plasmids into the TAP-tagged endogenous HisRS. We performed coimmunoprecipitation analysis using anti-GFP beads and tested the copurification of the endogenous, TAP-tagged HisRS. Analysis of the IP efficiency (by anti-GFP antibodies) revealed that both CD-GFP and ABD-GFP were isolated at similar levels. However, co-IP of HisRS-TAP was observed in the CD-GFP IP and not in the ABD-GFP IP (Fig 2D). Thus, consistent with crystallographic studies [31], the N-terminal region (which contains the CD), is the main dimerization region of HisRS. We conclude that the ABD domain is unlikely to associate with the emerging nascent chain and it interacts with mRNA in a non-cotranslational manner.

### HisRS interacts with a putative anticodon mimic within its mRNA

**Fig 3:**
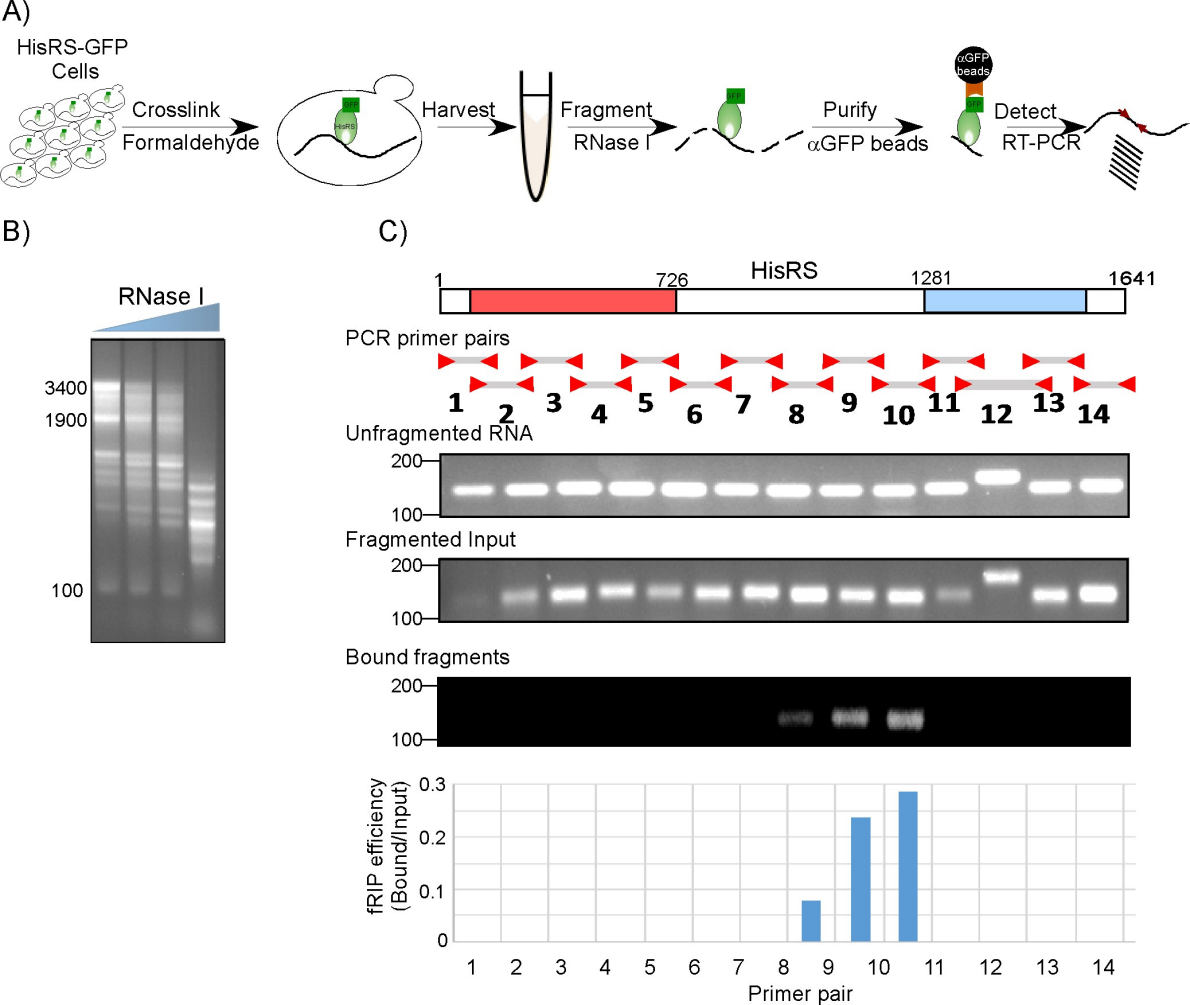
fRIP to map the RNA region bound by HisRS. A) Scheme of the fRIP protocol. Cell lysates of GFP-tagged HisRS strain are subjected to mild RNaseI treatment to generate fragments of about 200 nts. Fragmented input is mixed with αGFP beads (“GFP-Trap”) to isolate the HisRS-GFP bound fragments and wash away the unbound material. Bound fragments are identified by RT-PCR. B) RNaseI treatments were calibrated to determine conditions that yield RNA fragments of an average size of 200 nts. Cleavage products were run on an agarose gel, and migration sizes are indicated to the left. The highest RNaseI concentration was used for the fRIP protocol. C) Fourteen pairs of primers, generating products of similar length (except for pair 12), were designed to tile the entire HisRS-coding region. Semiquantitative PCR was done using this set to a cDNA from sample that was subjected to the entire procedure yet without RNaseI (Unfragmented RNA), sample that was treated with RNaseI yet was not mixed with the GFP beads (Fragmented Input) or fragmented sample that was subjected to GFP-Trap isolation (Bound fragments). Representative gels with samples from the same amplification cycle are presented. The histogram presents the ratio of signals between the Bound and Input for each fragment. Raw data is presented in S6 Table. fRIP, fragmentation RNA immunoprecipitation; GFP, green fluorescent protein; HisRS, histidyl-tRNA synthetase; RT-PCR, reverse transcription PCR.

We next wished to identify the site(s) along the mRNA that HisRS binds in vivo. To this end, we devised a fragmentation RIP procedure (fRIP) (Fig 3A). Extracts from cross-linked HisRS-GFP cells were subjected to mild RNaseI treatment to generate fragments of an average length of 200 nts (Fig 3B). Fragmented samples were then subjected to GFP-Trap isolation, which efficiently isolates HisRS-GFP with its associated fragments; thus, nonassociated fragments are washed away. Bound RNA was isolated and reverse transcribed with random hexamers. To identify the bound HisRS mRNA region, cDNA was subjected to PCR with 14 pairs of primers that tile the entire ORF region of HisRS (Fig 3C). These sets were spaced evenly along the gene to yield products of similar size (150 bp), hence lower variation in PCR efficiency (an exception is pair 12, which, due to Tm constraints, was planned to generate a slightly longer product). Control reactions were done with an unfragmented RNA sample (Fig 3C, upper panel) and with fragmented input prior to RNA isolation (Fig 3C, middle panel). The latter served to normalize differences in the efficiencies of RT-PCR reactions. As can be seen clearly in Fig 3C, bottom panel, fragment 10 (nucleotides 960–1,110 from the start codon) appears to be the most enriched by the fRIP procedure, hence likely to include the binding site. The signals of fragments 8 and 9 are probably due to a partial cleavage by the RNaseI, which leaves longer RNA fragments with the binding site. The lack of a complementing positive signal in fragment 11 is likely due to its low-PCR amplification efficiency, as evident from the “Fragmented Input” (Fig 3C).

**Fig 4:**
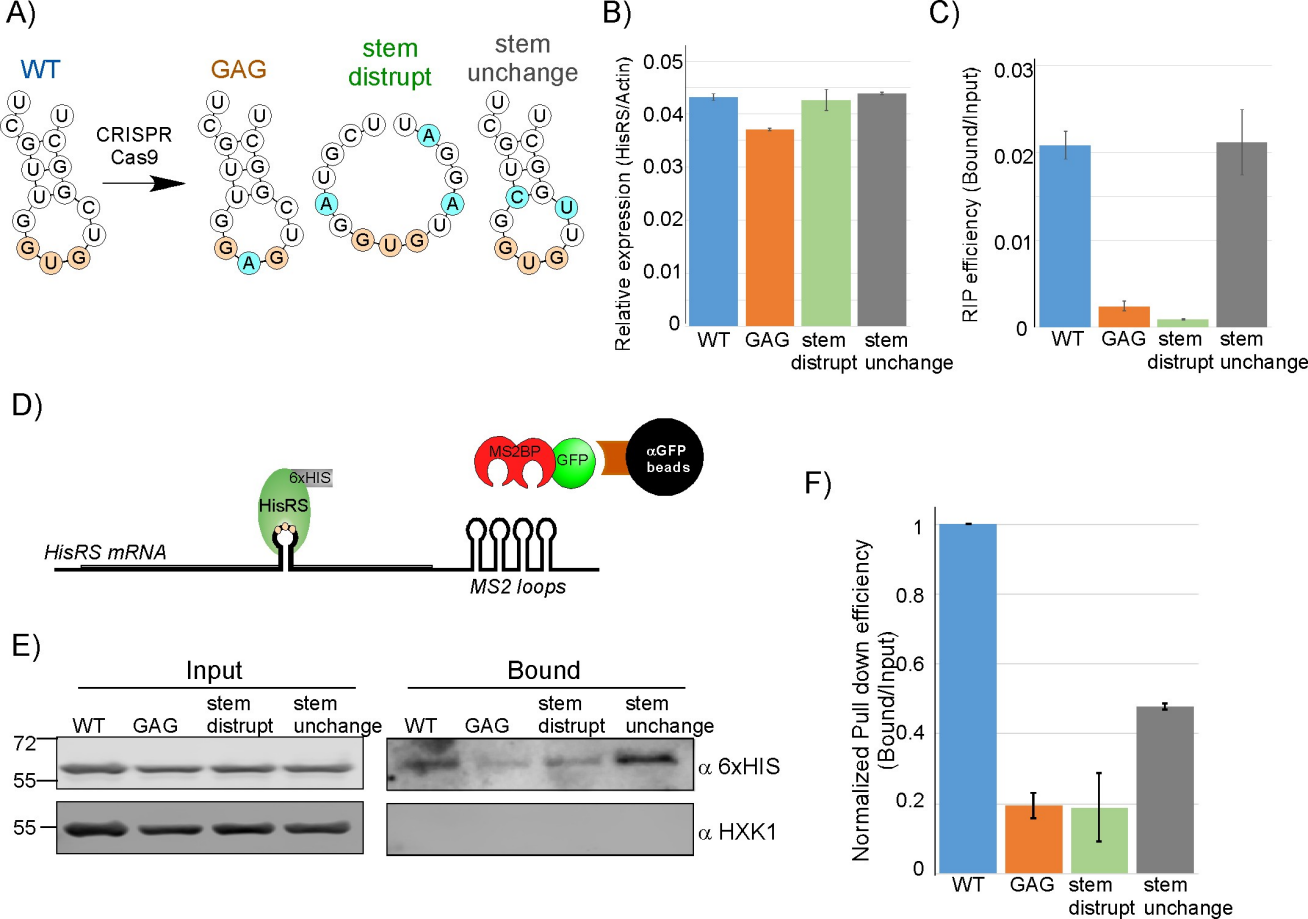
HisRS binds at an anticodon mimic. A) Predicted structures [32] of the GUG-containing stem-loop (predictions are for 15-nts window, starting at position 1094) and the introduced mutations. Blue circles indicate mutated bases. B) Steady-state mRNA levels measurements for the different HisRS variants. Input RNA samples were analyzed by RT-qPCR, using primers for HisRS mRNA or Actin. Histogram presents the SEM of three independent biological repeats, normalized to Actin levels. See S6 Table for raw data. C) RIP efficiency of the different HisRS variants. Histogram presents the ratio of the Bound to Input signal from two independent biological repeats measured by RT-qPCR. See S6 Table for raw data. Note that this RIP analysis was performed without a cross-linking step. D) RNA pull down assay. Each of the four anticodon mimic variants was tagged with 12 MS2-loops at its 3' UTR. Plasmid expressing MS2BP fused to GFP was also introduced into these cell. mRNAs bound by the MS2BP-GFP were isolated by anti-GFP beads, and association of HisRS protein was determined by western blot (HisRS was tagged at its C-terminus with 6xHIS). E) RNA pull down was performed to cells expressing the different variants, and protein samples from the input of the RNA pull down (Input) and the bound sample (Bound) were resolved in SDS-PAGE and subjected to western analysis with the indicated antibodies. F) Signals of Bound HisRS proteins were quantified by ImageJ, and divided by the signals in the Input in each strain. Histogram presents signals after normalization to the amount of pulled down HisRS-MS2 loops RNA (quantified by RT-qPCR) from two independent biological repeats. Error bars represent SEM. See S6 Table for raw data. GFP, green fluorescent protein; HisRS, histidyl-tRNA synthetase; MS2BP, MS2-binding protein; RIP, RNA immunoprecipitation; RT-qPCR, reverse transcription quantitative PCR.

Considering that the ABD was found to be important for binding (Fig 2C), we hypothesized that the RNA target site will be in an anticodon-like structure. In *S*. *cerevisiae*, HisRS recognizes only a single tRNA^His^, with a GUG in its anticodon loop (Fig 2B). RNA structural analysis [32] for fragment 10 region revealed a GUG within a stem-loop structure (note that the GUG is not in frame and is split between two codon: GGU [codon 366] and GUC [codon 367]) (Fig 4A). To determine whether this site is important for binding, we varied it by introducing two point mutations that are predicted to disrupt this element (Fig 4A): a single-base mutation that changes the anticodon-like sequence GUG into GAG (designated GAG) and triple-base mutation that predicts to disrupt the stem structure (Stem disrupt). A control, double mutation that is predicted not to affect the stem structure was also introduced (Stem unchanged) (Fig 4A). All mutations were introduced into the genomic loci by clustered regularly interspaced short palindromic repeats/ CRISPR-associated protein 9 (CRISPR/Cas9) [33,34], and Sanger sequencing confirmed that no other nucleotide in the gene was affected. Importantly, all the introduced mutations were silent and therefore did not affect the encoded HisRS protein. RT-qPCR analysis revealed minor changes in the abundance of the transcript in any of the mutants (Fig 4B). However, RIP followed by RT-qPCR revealed a significant decrease in the association of HisRS with the GAG-containing transcript and with the transcript with disrupted stem. Importantly, the mutations that did not change the stem did not affect the association with HisRS (Fig 4C).

To further substantiate the validity of this putative anticodon mimic, we designed a reciprocal experiment in which the mRNA was pulled down and the levels of its associated HisRS protein was determined. For that, each of the four mRNA variant (wild-type [WT], GAG mutation, stem disruption, and stem unchanged) was tagged at its 3' UTR with multiple MS2 loops and expressed together with a plasmid expressing MS2-binding protein fused to GFP (MS2BP-GFP). Transcripts were isolated on GFP-Trap columns by virtue of their association with the MS2BP-GFP fusion (Fig 4D). Western analysis for the levels of associated HisRS (tagged with 6xHIS) are presented in Fig 4E. These signals were quantified and normalized to the amounts of pulled-down HisRS-MS2 loops mRNAs (measured by a parallel RT-qPCR) to account for variation in pull down efficiencies (Fig 4F). The results are in clear concordance with the RIP assay. Namely, high association of HisRS with the WT transcript, reduced association with the GAG and stem disruption, and higher association with the unchanged-stem mutant transcript (Fig 4F). Together, these results show that HisRS binds its mRNA and validate the importance of an anticodon-like element for binding.

### Binding to the putative anticodon GUG represses HisRS translation

**Fig 5:**
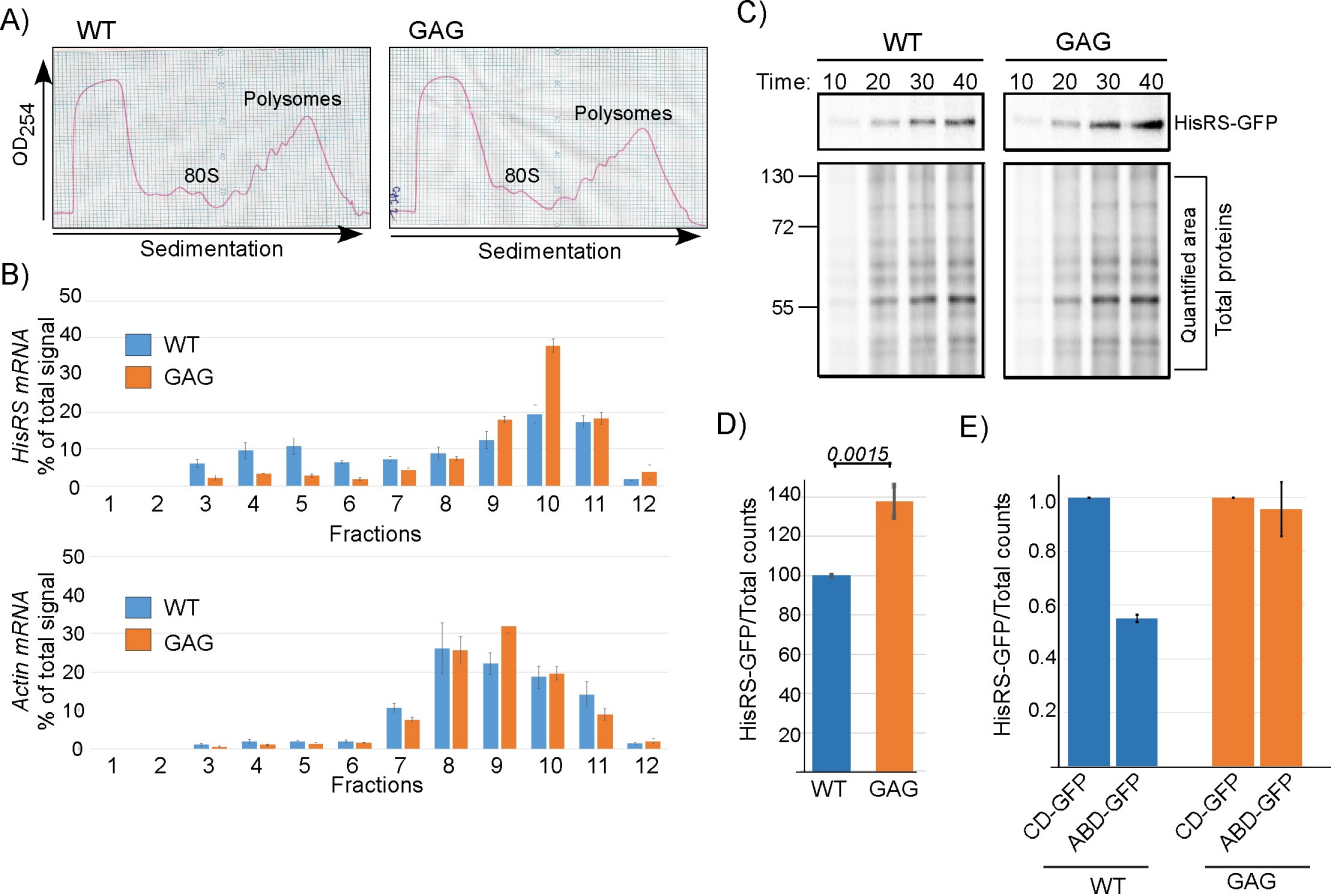
Binding of HisRS to the anticodon mimic represses its translation. A) Cells expressing either WT HisRS or GAG mutation were subjected to polysomal fractionation on a sucrose gradient. OD_254_ profiles of the WT and GAG reveal similar polysomal profiles. B) Twelve fractions were collected along the gradients and subjected to northern analysis with either HisRS probe or Actin probe. Histograms presents the quantification of the HisRS mRNA signal (top) and the Actin mRNA signal (bottom) from three independent biological repeats. Error bars represent the standard deviation of percent of signal per fraction. See S6 Table for raw data. C) Cells were pulse labeled with ^35^S-met and samples were collected at the indicated times. Total protein sample was set aside, and HisRS-GFP was isolated from the remainder by IP. Autoradiogram presents HisRS-GFP signals and Total proteins signals (with the quantified region indicated) from both strains. D) Cells were subjected to a 30-min pulse, and labeled HisRS-GFP was isolated by immunoprecipitation as in C. Signals were normalized to the Total proteins signal. Results are from four independent biological repeats. Error bars represent SEM, and *P* value was calculated by the dependent samples one-sided *t* test. See S6 Table for raw data. E) Plasmids expressing the CD-GFP or ABD-GFP were introduced into cells endogenously expressing either the WT or GAG variants of HisRS. Cells were subjected to a 30-min pulse, followed by HisRS immunoisolation as in C. Results are from two independent biological repeats, and error bars are SEM. See S6 Table for raw data. ABD, anticodon-binding domain; CD, catalytic domain; GFP, green fluorescent protein; HisRS, histidyl-tRNA synthetase; IP, immunoprecipitation; OD_254,_ optical density; WT, wild-type.

HisRS binding to its own mRNA might play a post-transcriptional regulatory role. Since transcript levels of the WT and the mutated variants are similar (Fig 4B), we hypothesized that impact is at the translation level. To address this, we performed polysomal association analysis for the WT and anticodon GAG mutation variant. optical density at 254 nm (OD_254_) profile of polysomal distribution in sucrose gradients did not reveal any general difference between the WT and GAG-containing strains (Fig 5A). Gradients were fractionated and RNA was isolated from each fraction and subjected to northern analysis with probes recognizing the HisRS mRNA or Actin control. An increase in ribosomal association (in particular fractions 9 and 10) and a decrease in nonribosomal (fractions 3–6) is observed for the anticodon GAG variant compared to the WT transcript. No significant change is observed for Actin (Fig 5B). We also measured protein synthesis by pulse labeling of proteins by adding L- [^35^S]-methionine (^35^S-met) to the growth medium of both strains. Aliquots were collected at different time points after addition, lysed, and subjected to PAGE autoradiography either directly (Total proteins) or after isolation of HisRS-GFP by GFP-Trap beads (Fig 5C). Overall protein synthesis appeared similar for both strains (Fig 5C), indicating that the silent mutation did not lead to general effects on translation. However, the synthesis of the GAG-containing HisRS appeared higher after 30-min or longer labeling (Fig 5C). Biological repeats of a 30-min pulse revealed a 40% increase in HisRS protein that is made from the GAG variant (Fig 5D). We note that the nucleotide change that was introduced (U to A) changes the wobble position of a GGU codon into GGA. GGA has a lower codon bias index than GGU; hence, it is unlikely that the increased translation of the GAG variant is due to a change in codon bias. These results therefore suggest that the increased translation of HisRS is due to relief of a translation inhibition imposed by binding of HisRS to the GUG region.

To explore if inhibition can occur in *trans*, we introduced plasmids expressing ABD-GFP or CD-GFP into cells either endogenously expressing the WT HisRS or the GAG variant. Cells were subjected to pulse labeling, followed by immunoisolation of the endogenously expressed HisRS. As can be seen in Fig 5E, introducing the ABD-GFP repressed the synthesis of HisRS compared to CD-GFP. Importantly, the impact of ABD-GFP on translation was not detected in the GAG HisRS variant. Thus, interaction between the ABD and the anticodon mimic is critical for inhibition.

### Binding regulates HisRS translation in response to tRNA^His^ levels

**Fig 6:**
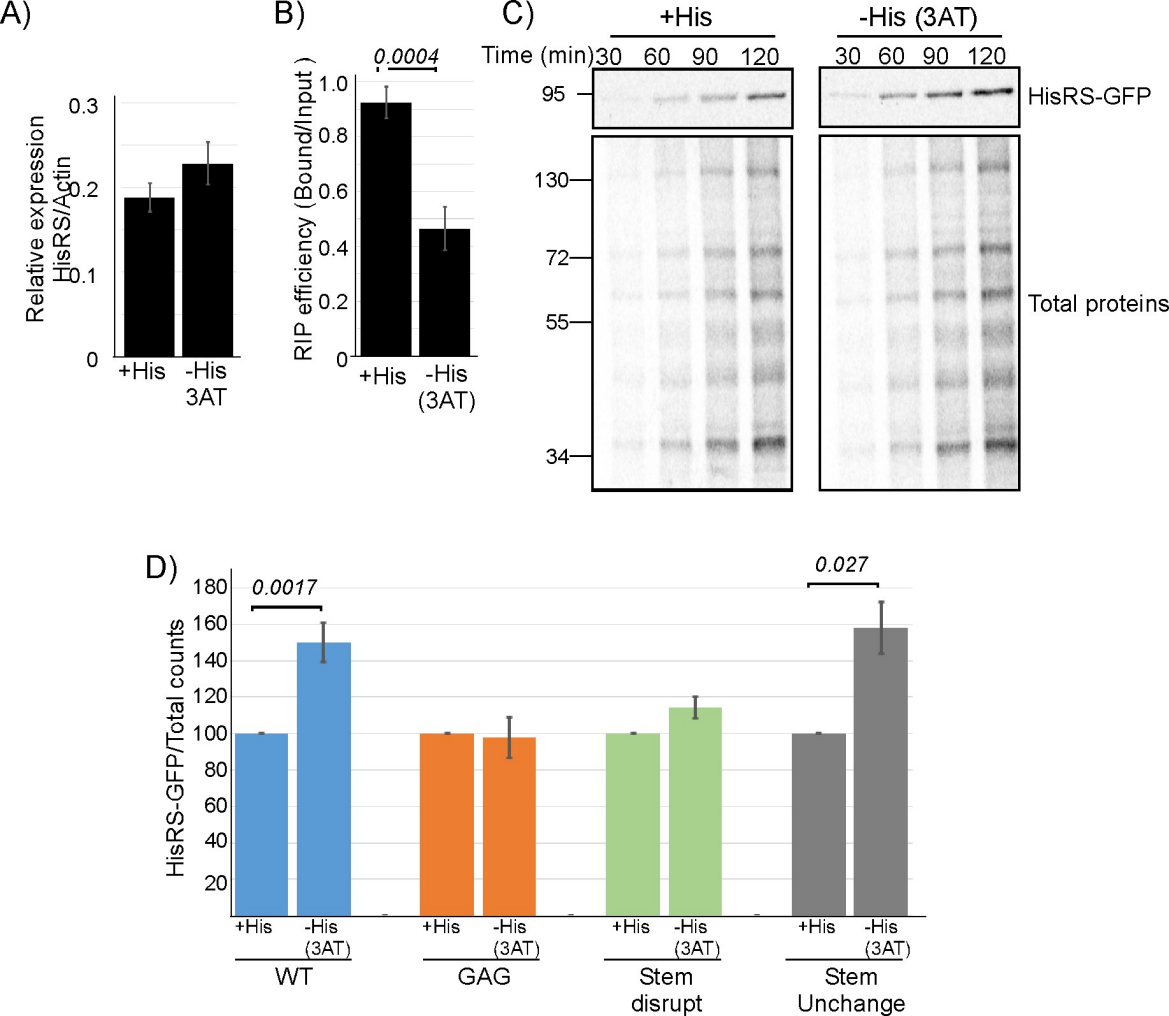
Anticodon mimic–dependent regulation of mRNA binding and HisRS protein synthesis. A–B) Cells were grown either in the presence of histidine (+His) or without histidine and with 100-mM 3AT (−His) for 1 hour and subjected to GFP-Trap mRNA isolation. A) RT-qPCR analysis of steady-state (Input) HisRS mRNA levels, normalized to Actin levels. B) RIP efficiency (ratio of Bound to Input), as measured by RT-qPCR. Error bars are SEM from two independent biological repeats, and *P* value was calculated by the dependent samples one-sided *t* test. See S6 Table for raw data. C) Cells were subjected to 3AT treatment as in A and concomitantly pulsed with ^35^S-met for the indicated times. Total proteins' sample was set aside, and labeled HisRS was isolated from the remainder by IP. Autoradiographs present the signals of HisRS and Total proteins samples. D) Cells expressing WT or anticodon mimic variants were subjected to a 90-min pulse concomitant with His depletion. Labeled HisRS-GFP was isolated by immunoprecipitation as in C. Histograms present the quantification of the HisRS signals, normalized to the Total proteins signal. Results are from four (for the WT variant) and two (for the anticodon mimic variants) independent biological repeats. Error bars are SEM and *P* value was calculated by the dependent samples one-sided *t* test. See S6 Table for raw data. 3AT, 3 aminotriazole; GFP, green fluorescent protein; HisRS, histidyl-tRNA synthetase; IP, immunoprecipitation; RIP, RNA immunoprecipitation; RT-qPCR, reverse transcription quantitative PCR; WT, wild-type.

The association of HisRS with its own mRNA through a tRNA^His^ anticodon mimic may offer a regulatory mechanism, which coordinates mRNA translation with tRNA availability. To address this in vivo, we subjected cells to starvation of histidine (by the addition of 3 aminotriazole [3AT]), a stress condition known to increase the levels of uncharged tRNA^His^ [35,36]. RT-qPCR analysis of expression levels revealed a small increase in steady-state mRNA levels of HisRS, suggesting some regulation at the transcription level (Fig 6A). Nevertheless, binding efficiency of HisRS to the mRNA is significantly reduced upon this treatment (Fig 6B). Furthermore, protein synthesis measurements by ^35^S-met pulse labeling revealed that HisRS protein synthesis rate is significantly higher upon histidine depletion (Fig 6C). Biological replicates for a 90-min pulse time revealed a 50% increase in synthesis (Fig 6D). Importantly, this translation enhancement is diminished in the GAG and stem disruption mutants, yet apparent in the unchanged stem variant of HisRS (Fig 6D). Thus, binding to the putative anticodon mimic is critical for translation regulation.

**Fig 7:**
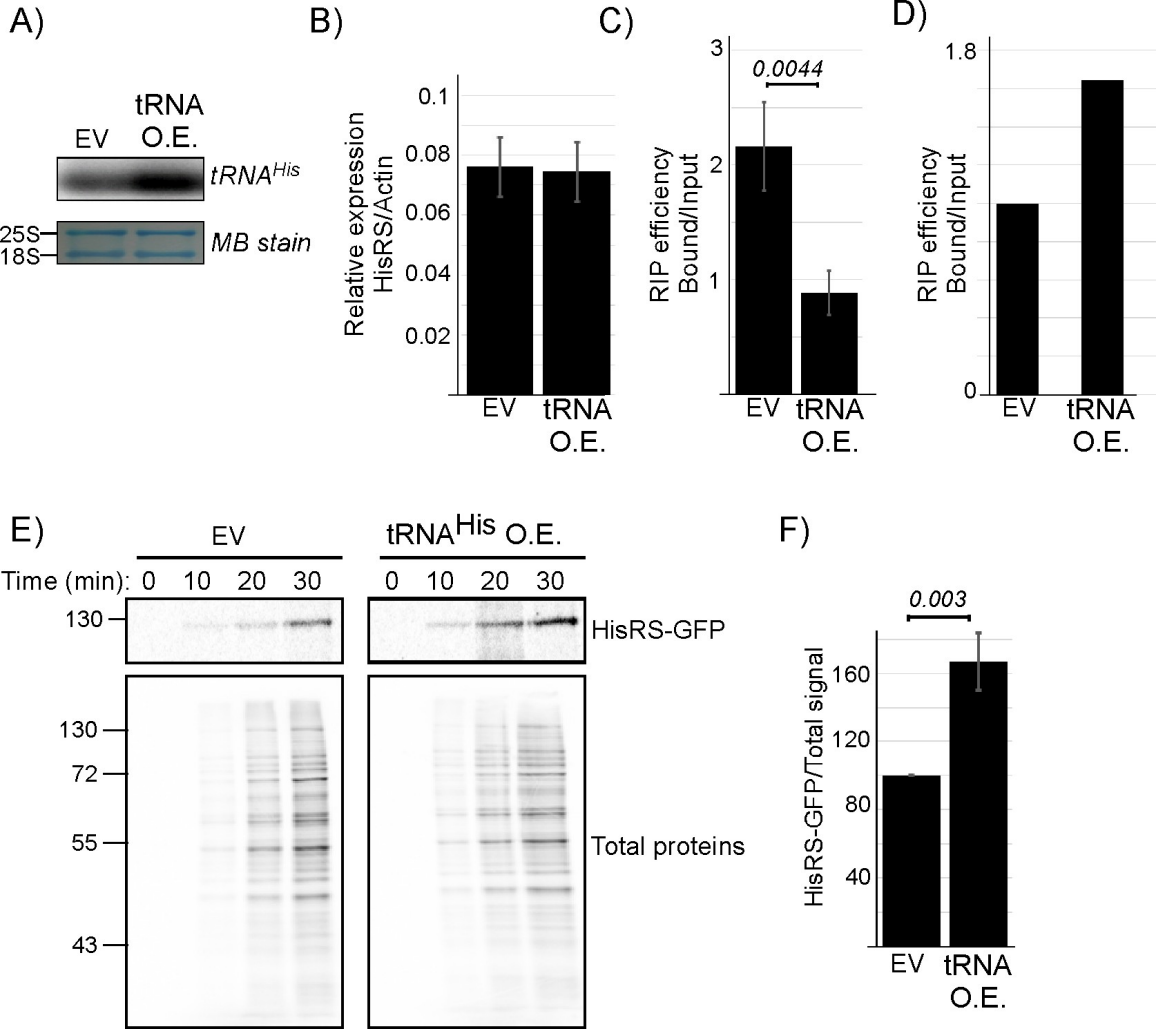
tRNA^His^ levels affect mRNA binding and HisRS protein synthesis. Plasmid expressing tRNA^His^ (tRNA^His^ OE) or an EV control were introduced into HisRS-GFP strain. A) Northern analysis of tRNA^His^ expression levels using a probe recognizing tRNA^His^. Lower panel is a MB stain of the same blot as loading control. B) RT-qPCR analysis of HisRS mRNA levels normalized to Actin in cells transfected as indicated. Results are from three independent biological repeats. See S6 Table for raw data. C) RT-qPCR analysis of mRNA binding upon tRNA^His^ overexpression. Cells were subjected to RIP, followed by RT-qPCR. The histogram presents the quantification of two independent biological repeats. *P* value was calculated by dependent samples one-sided *t* test. See S6 Table for raw data. D) RNA samples from RIP procedure were subjected to northern analysis with a tRNA^His^ probe. The histogram presents the quantification of signals, indicating that tRNA^His^ association increases upon tRNA^His^ overexpression. See S6 Table for raw data. E) Cells overexpressing tRNA^His^ or an EV were pulse-labeled with ^35^S-met, and samples were collected at the indicated times. Total proteins sample was set aside, and HisRS-GFP was isolated from the remainder by IP. Total and IP samples were resolved by SDS-PAGE. The upper autoradiogram presents HisRS-GFP signals from both strains and the bottom presents the Total proteins signals. F) Cells were subjected to a 30-min pulse, and labeled HisRS-GFP was isolated by immunoprecipitation as in D. Signals were normalized to the Total proteins signal. Results are from four independent biological repeats, normalized to the Total proteins signal. Error bars represent SEM, and *P* value was calculated by the dependent samples one-sided *t* test. See S6 Table for raw data. EV, empty vector; GFP, green fluorescent protein; HisRS, histidyl-tRNA synthetase; IP, immunoprecipitation; MB, methylene blue; OE, overexpression; RIP, RNA immunoprecipiation; RT-qPCR, reverse transcription quantitative PCR.

To pinpoint the observed changes to an increase in tRNA^His^ levels, we introduced into HisRS-GFP strain a plasmid that overexpresses tRNA^His^ about 5-fold higher than normal (Fig 7A). tRNA overexpression did not affect steady-state levels of HisRS mRNA (Fig 7B). Importantly, HisRS RIP analysis followed by RT-qPCR revealed a strong decrease in mRNA self-association (Fig 7C), which occurred concomitantly with an increase in tRNA association (Fig 7D). Analysis of protein synthesis by ^35^S-met pulse labeling revealed that the decreased mRNA binding coincides with increased HisRS protein synthesis, without an effect on overall translation (Fig 7E and 7F). These results indicate a direct linkage between increased tRNA acylation demands, decreased mRNA binding and increased HisRS protein synthesis.

## Discussion

### aaRSs bind their own mRNA

The data presented herein reveals an autogenous association between every tested aaRS and its mRNA. This implies a general post-transcriptional autoregulatory process for this family of RBPs. Other RBPs are also known to be subjected to autoregulation in *S*. *cerevisiae*. Some ribosomal proteins were found to bind their own mRNA [37–39]. Here, binding appeared to affect the splicing of these mRNAs and thereby regulate their expression. Self-association appears also for bona fide mRNA-binding proteins; the mRNA targets of 46 mRBPs were characterized [40], and 18 of them appeared to be highly associated with their own mRNA [41]. Thus, we propose that self-association with their mRNA is an abundant feature of the RNA-binding proteome.

### Possible expression-regulation mechanisms

Post-transcriptional autoregulation by RBPs may occur at various stages. As indicated above, ribosomal proteins usually regulate their splicing process. In the case of AspRS, regulation was shown to occur at the step of mRNA export from the nucleus [42]. Our work indicates regulation by RBP at the step of translation. The exact mechanisms of translation regulation are yet to be explored. One possibility is that HisRS binding affects translation initiation and ribosomal loading. Our polysomal data (Fig 5) reveal that reduced mRNA binding by HisRS results in a decrease in ribosome-free mRNA and an increase in polysomal mRNA. Such changes are consistent with a role in translation initiation. On the other hand, the binding site of HisRS is within its coding region, suggesting regulation at the elongation step of translation. HisRS binding may slow ribosomal transit at this region, as was shown to occur upon binding of the neuronal RBP fragile X mental retardation 1 (FMRP) to some mRNAs [43]. Yet analysis of Ribo-seq mapping results in the genome-wide information on protein synthesis (GWIPS)-vis site did not reveal any accumulation of ribosomes upstream to the anticodon mimic. Furthermore, 3AT treatment did not reveal any gross changes in ribosome footprint on this area of HisRS mRNA [44]. An alternative mode of elongation regulation may involve association of the aaRS with the nascent protein chains as they emerge from ribosomes (i.e., cotranslational). Such mode of regulation was shown recently to coordinate the assembly of protein complexes [28]. In the case of MetRS:Arc1:GluRS, our data support this model and expand it. Specifically, the reduction in GluRS mRNA levels upon deletion of Arc1 from the MetRS-GFP strain indicates that complex formation is important for partner mRNA isolation. However, the fact that self-association is hardly affected from Arc1 deletion suggests an additional interaction mode that is independent of complex assembly. Together, we suggest that MetRS and GluRS regulate each other's expression in a cotranslational, complex-dependent manner, yet regulate their own expression through direct binding to their mRNA and not through their nascent chain. Interestingly, none of the proteins that were presented in detail in [28] (Fas1, Fas2, GluRS, MetRS, Arc1, Trp2, Trp3, Pfk1, Pfk2) appeared to associate with its own nascent chain. We therefore propose that a complete model for complex assembly entails, in addition to association with partners’ nascent chain, also direct association with self mRNA. Notably, Pfk2 was also shown to bind its own mRNA [10], suggesting that this occurs also in the Phosphofructokinase complex.

### Autogenous regulation of expression by tRNA mimic

The data presented here reveal a post-transcriptional autogenous regulation mechanism that is suggested to utilize similar RNA moieties. Such a mechanism was proposed years ago for the *E*. *coli* HisRS by Ames B. and colleagues, following an observation of a tRNA^His^-like structure at the operator region [45]. The predicted structure of the element that we identified herein shows some resemblance to the anticodon moiety of tRNA^His^, and our mutagenesis results are consistent with the structure prediction. Further support for similarity with tRNA^His^ anticodon loop is derived from the fact that HisRS binds this element through the same domain that it binds the tRNA^His^ anticodon (i.e., the ABD). It should be noted, however, that due to the presence of the putative anticodon mimic within the coding region, we were limited in our ability to perform comprehensive mutagenesis analysis without affecting the encoded protein. Thus, an unequivocal confirmation of structural mimicry will necessitate other means. For example, in vitro interaction assays with purified components or introducing the putative element upstream to a reporter gene, followed by mutagenesis analysis of predicted structures. Such approaches were applied in studies of the *E*. *coli* ThrRS and revealed an operator region that post-transcriptionally controls ThrRS expression [46] through autogenous binding to a tRNA-like region within it [22,47,48]. Protein binding blocks ribosomes’ association and thereby represses ThrRS translation initiation. tRNA-like structures were also found to be important for *S*. *cerevisiae* AspRS self-association [23,49]. Thus, binding through RNA mimicry might be a general property of aaRS. Binding through RNA structural mimicry is actually well-established for most ribosomal proteins in *E*. *coli*. Structural elements within the operators of *E*. *coli* ribosomal protein genes resemble the binding sites of these proteins within rRNA [50,51]. Thus, when ribosomal proteins are at access to rRNA (i.e., are not bound to rRNA), they bind these operators and repress ribosome binding. Together, RNA mimicry might be a general mechanism by which RBPs coordinate different cellular processes.

### Regulation by levels of uncharged tRNA

**Fig 8:**
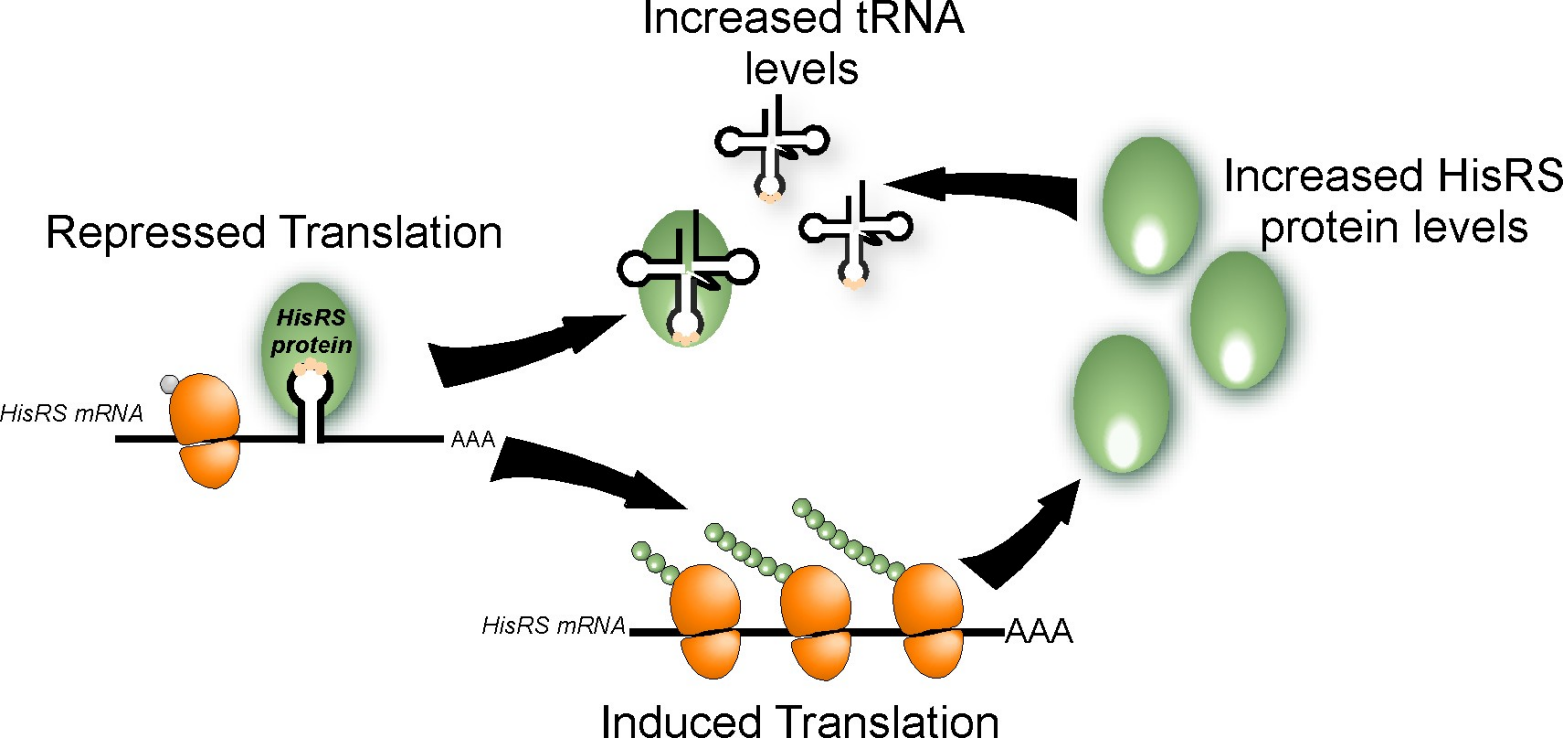
Model for aaRS-mediated regulation. HisRS binds its mRNA through an anticodon-like element and inhibits translation. Upon an increase in uncharged tRNA levels, binding shifts to the tRNA and translation inhibition is relieved. This leads to increased HisRS protein levels, which are necessary to charge the excess of tRNA. aaRS, aminoacyl-tRNA synthetase; HisRS, histidyl-tRNA synthetase.

We presented here evidence that changes in the amounts of tRNA^His^ affect the translation of HisRS. Specifically, depletion of histidine (Fig 6) and overexpression of tRNA^His^ (Fig 7) led to an increased synthesis of HisRS. Increased levels of uncharged tRNA^His^ induce broad changes in gene expression through activation of the amino acids starvation pathway. The activation mechanism includes binding of tRNA^His^ to a domain in Gcn2 that is similar to HisRS, leading to activation of the kinase domain within the protein [52,53]. Gcn2 in turn activates the synthesis of the transcription regulator Gcn4 and thereby allows proper response to cellular needs. Our model for HisRS activation (Fig 8) suggests a direct mechanism, in which tRNA^His^ binds directly to HisRS, and relieves the inhibition it imposes on translation. Thus, excess of uncharged tRNA^His^ may operate in two ways: binding to Gcn2 to activate the general amino acid starvation pathway and binding to HisRS to induce its translation. The increased HisRS synthesis leads to higher amounts of the enzyme, which are necessary to account for the increased charging demands.

## Conclusions

Overall, we found that in vivo, aaRSs preferentially bind their own mRNA. These proteins are therefore tRNA and mRNA binders. This dual binding property allows coordination between tRNA charging and mRNA translation and, more generally, between translation and gene expression. We also show that for HisRS, binding occurs through a putative anticodon mimic. As anticodon recognition is central for tRNA binding by most aaRSs, we propose that recognition through anticodon-like elements will be a prevalent mode of interaction by most aaRS. Single-nucleotide resolution mapping strategies are necessary to confirm this and, in particular, methodologies that combine structural analysis. Such approaches are especially necessary in cases in which the aaRS interacts with multiple anticodons and therefore is likely to recognize various elements along the mRNA. Further, we show that mRNA binding leads to translation inhibition, and the mechanisms by which binding impacts ribosomal association are yet to be uncovered. Finally, we demonstrated that regulation involves competitive association of the aaRS with its target tRNA and mRNA. These results reveal a new mechanism for communicating gene expression with tRNA charging (Fig 8) in which a single RBP coordinates distinct cellular processes.

## Materials and methods

### Yeast strains, plasmids, and growth conditions

The following yeast strains were used: endogenously TAP-tagged (TAP-His3MX) *MES1* (MetRS), *GUS1* (GluRS), *VAS1* (ValRS), and *HTS1* (HisRS) were all in the following genetic background (ATCC 201388: *MATa his3Δ1 leu2Δ0 met15Δ0 ura3Δ0*) [54], and endogenously GFP-tagged (GFP [S65T]-His3MX) *GLN4* (GlnRS), and *HTS1* (HisRS) were all on the following background (ATCC 201388: *MATa his3Δ1 leu2Δ0 met15Δ0 ura3Δ0*) [55]. Proper fusion and expression of the tags were verified by PCR and western analysis. Knockout of *ARC1* was done in the MetRS-TAP background by homologous recombination using a *LEU2* cassette amplified from pRS405, as describe in [56].

Plasmids expressing CD-GFP and ABD-GFP were generated by PCR amplification of nts 1–1,281 (for CD) and 1,281–1,641 (for ABD) and cloning upstream to enhanced GFP (eGFP). The HisRS domain-eGFP fragment was cloned into the vector backbone of pAD-GAL4 (Stratagene) by replacing the GAL4-AD region. Plasmids were introduced into BY4741 cells and grown in SC with the appropriate drop outs. Western analysis confirmed similar expression of the two fusion proteins.

tRNA^His^ expression plasmid (2 μ, *LEU2*), containing the tRNA^His^ (GUG) (tH[GUG]G2) mature sequence [57] and empty vector control, were introduced into HisRS-GFP strain and grown in SC with the appropriate dropout.

### RIP

Strains (either TAP or GFP tagged) were grown in YPD to mid logarithmic phase and subjected to cross-linking by addition of formaldehyde (0.05% final concentration) for 10 min at room temp. Cross-linking was terminated with 0.125 M glycine for 3 min, and cells were lysed using bead beater in Buffer B (20 mM Tris-HCl [pH 7.5], 140 mM NaCl, 0.1% NP40, 0.1% SDS, 0.5 mM EDTA, 1 mM DTT, 2 mM PMSF, 10 μg/ml Leupeptin, 14 μg/ml Pepstatin, 0.02 U/μl RQ1 RNase-free DNase (Promega), 0.24 U/μl RiboLock RNase Inhibitor [Thermo Scientific]). Lysate was cleared by centrifugation at 10,600 g for 10 min at 4°C. For isolation of TAP-tagged proteins, lysates were loaded on proteinA-Sepharose beads (GE Healthcare 17-0969-01) and rotated at 4°C for 2 hr. Samples were washed four times in Buffer C (20 mM Tris-HCl [pH 7.5], 1 M NaCl, 0.5% NP40, 0.1% SDS, 0.5 mM EDTA, 0.5 mM DTT, 0.01 U/μl RiboLock RNase Inhibitor [Thermo Scientific]) and subjected to cleavage with 80 U of TEV (Invitrogen 12575–015) for 2 hr at 16°C in TEV buffer. Cross-linking was reversed by heating at 65°C for 2 hr in reverse cross-linking buffer (50 mM Tris-HCl [pH 7.5], 5 mM EDTA, 10 mM DTT, 1% SDS, 0.1 U/μl RiboLock RNase Inhibitor [Thermo Scientific]), and RNA was precipitated after phenol:choloroform extraction. For isolation of GFP-tagged proteins, lysates were loaded on GFP-Trap_A (ChromoTek) and rotated at 4°C for 2 hr. Samples were washed four times in Buffer C and eluted by 0.2 M Glycine buffer (pH 2.5). Cross-linking reversal and RNA isolation was done as described above for TAP-tagged sample.

### UV–RNA cross-linking immunoprecipitation (CLIP)

GFP-tagged strains were grown in YPD to mid logarithmic phase, subjected to UV cross-linking (1.2 J/cm^2^ of 254 nm) on ice. Cells were lysed using bead beater in buffer B and immunoprecipitation was conducted as described above. Cross-linking was reversed by 0.8 U/ μl proteinase K (NEB P8107S) at 50°C for 1 hr in PK Buffer (100 mM Tris [pH 7.4], 50 mM NaCl, 0.1% SDS, 10 mM EDTA), and RNA was precipitated after phenol:choloroform extraction.

### RNA quantification methods

RNA samples were subjected to RNA-seq at the Technion Genome Center. Libraries were prepared using TruSeq RNA Library Prep Kit v2 (Illumina, California, United States of America) according to the manufacturer's instructions. The polyA selection step was excluded for the Bound samples to minimize losses. All samples were sequenced on Illumina HiSeq 2500 platform, yielding 10 to 18 million reads per sample. The reads were mapped to the S288c Saccharomyces cerevisiae version R64-2-1 genome using Tophat2 version 2.1.0 with up to three mismatches allowed per read. Only uniquely mapped reads were counted to genes using HTSeq-count package version 0.6.1.

For semiquantitative PCR, cDNA was prepared from 500 ng RNA by High-Capacity cDNA Reverse Transcription Kit (Maxima) according to the manufacturer’s instructions, and subjected to PCR with gene-specific primers (S5 Table). Aliquots were set aside every five cycles, run on agarose gel, and stained with ethidium bromide. For RT-qPCR, RNA was reverse transcribed using High-Capacity cDNA Reverse Transcription Kit (Maxima) according to the manufacturer’s instructions. Gene-specific transcription levels were determined in a 25-μl reaction volume in triplicate using qPCR master mix (Applied Biosystem cat. 4367659), following the manufacturer's instructions using primers for the indicated genes (S5 Table). Results were analyzed with 7500 software v2.0.6 program.

### RNA pull-down

Strains expressing HisRS mRNA variants (WT, GAG, Stem disrupt, and Stem unchange) tagged at the 3' UTR with multiple MS2 loops and expressing pCP-GFP [58] were grown to logarithmic phase (OD600 0.8–1) in 500 ml. For RNA “Input” sample, 10 ml were set aside, and RNA were extracted by the hot phenol method [59]. The remainder of cells (490 ml) were subjected to cross-linking by addition of formaldehyde (0.05% final concentration) in PBS and slowly rotated at room temperature for 10 min to cross-link protein–RNA complexes. 0.125 M Glycine was added to stop the cross-linking reaction. Cells were then suspended in 1 ml cold buffer B (with 5 mg/ml Heparin final concentration). Cells were lysed and cleared as in RIP method. Aliquot (1/20 of the lysate) were set aside for proteins' “Input” sample. Lysates were loaded on GFP-Trap_A (ChromoTek) and rotated at 4°C for 2 hr. Samples were washed four times in wash buffer (20 mM Tris-HCl [pH 7.5], 0.5 M NaCl, 0.5% NP40, 0.1% SDS, 0.5 mM EDTA, 0.5 mM DTT, 0.01 U/μl RiboLock RNase Inhibitor [Thermo Scientific]). Proteins were eluted from 80% of the beads with LSB and resolved on 10% PAGE. RNA was extracted from 20% of the beads by 0.2 M Glycine buffer (pH 2.5). Cross-linking was reversed by heating at 65°C for 2 hr in reverse cross-linking buffer, and RNA was precipitated after phenol:choloroform extraction.

### fRIP

HisRS-GFP strain was grown in YPD to mid logarithmic phase and subjected to cross-linking by addition of formaldehyde (0.1% final concentration) for 10 min at room temp. Cross-linking was terminated with 0.125 M glycine for 3 min, and cells were lysed in Buffer B. Lysate was cleared by centrifugation for 10 min at 10,600 g at 4°C and fragmented by 0.2 U/μl RNase I (LifeTech, AM2295), with shaking for 4 min at 37°C. Samples were loaded on GFP-Trap_A (ChromoTek) for 2 hr at 4°C, and washed four times in Buffer C. Proteins were eluted by 0.2 M Glycine buffer (pH 2.5). Cross-linking was reversed by heating at 65°C for 16 hr in reverse cross-linking buffer and RNA was precipitated following phenol:choloroform extraction. For semiquantitative PCR, cDNA was prepared by High-Capacity cDNA Reverse Transcription Kit (Maxima) according to the manufacturer’s instructions and subjected to 14 PCR reactions with specific primers that designed to cover the entire HisRS transcript. Aliquots were set aside every five cycles, run on agarose gel, and stained with ethidium bromide.

### CRISPR/Cas9 point mutagenesis

Point mutations by the CRISPR/Cas9 were introduced according to protocol [33]. Briefly, plasmids expressing guideRNA recognizing the mutation region in bRA66 (Addgene #100952) backbone was transformed into HisRS-GFP strain together with double-stranded 80-bp DNA fragment that contains the desired mutation at its center. Transformants were grown on YP-Gal Hygromycin (200 μg/ml) plate to induce Cas9 expression. Control transformation without the 80-bp DNA fragment did not yield any viable colony, indicating that the double-strand break induced by the guideRNA is lethal. Viable clones from the transformation with the 80 bp fragment were isolated, and the HisRS gene was sequenced. All included only a single change at the desired site. The exact positions of mutations are GAG mutant: T1101A; Stem disrupt: T1098A, C1104A, C1107A; Stem unchanged: T1098C, C1104T.

### MS2 loops tagging of HisRS

Plasmid expressing guideRNA recognizing GFP (S65T) sequence in bRA66 (Addgene #100952) backbone was transformed into either HisRS-GFP WT, GAG, Stem disrupt, or Stem unchanged strains together with PCR product amplified from pSL-MS2-12X (Addgene #27119) using HisRS MS2L F and HisRS MS2L primers (S5 Table). Note that the MS2L F primer also includes 6XHIS, thus adds 6XHIS in frame to HisRS before its stop codon. Transformants were grown on YP-Gal Hygromycin (200 μg/ml) plate to induce Cas9 expression. Clones were isolated, and the tagging at the genomic locus were validated by sequencing.

### ^35^S methionine pulse labeling

Cells (10 ml) were grown to mid-log phase in SC, washed, and resuspended in 10 ml SC-met. Growth was continued for another 5 min, ^35^S methionine (NEG709A005MC, PerkinElmer) was added, and 2-ml aliquots were set into cycloheximide (100 μg/ml final concentration) at the indicated times. Labeled HisRS-GFP was immunoprecipitated with GFP-Trap_A (ChromoTek) by incubation at 4°C for 2 hr and washed four times in Buffer C. Proteins were eluted from the beads with LSB, resolved on 10% PAGE, and exposed to phosphorimager. Radioactive signals were quantified by ImageJ.

### Polysomal profiles

Polysome analysis was done as described [60]. Briefly, 100-ml cells were grown to mid-log phase, cycloheximide was added, and cells were harvested in Lysis buffer (20 mM Tris/HCl [pH 7.4], 140 mM KCl,1.5 mM MgCl_2_, 0.5 mM DTT, 100 μg/ml cycloheximide,1 mg/ml heparin, 1% Triton X-100). Lysates were cleared by 10 min centrifugation at 9,000 g, and loaded on 10%–50% linear sucrose gradient. Polysomal complexes were resolved by 160 min centrifugation at 35,000 rpm in SW41 rotor and separated into 12 fractions with ISCO UA6 device. RNA was extracted from each fraction and analyzed by northern blot with probes for HisRS or Actin mRNAs [61].

## Supporting information

S1 Fig RIP is highly specific. Yeast strains expressing tagged aaRSs were subjected to RIP using the TAP or GFP-Trap beads. Protein samples from the total cellular lysate (Input), unbound FT, LW step, and the eluted fraction (Bound) were resolved on PAGE followed by western analysis with the indicated antibodies (A) or stained with Silver stain (B). Equivalent amounts were loaded except of the Bound sample that was three times larger. Size markers are shown to the left. aaRS, aminoacyl-tRNA synthetase; FT, flow-through; GFP, green fluorescent protein; LW, last washing; RIP, RNA immunoprecipitation; TAP, Tandem Affinity Purification.
(PDF)

S2 Fig mRNAs bound by aaRSs. Venn diagrams were generated from the mRNAs that appeared within the 1.5 IQR region among all strains that were subjected to the TAP protocol (A), GFP-Trap protocol (B) and for HisRS tagged with either TAP of GFP (C). Shared genes or GO terms are indicated. aaRS, aminoacyl-tRNA synthetase; GFP, green fluorescent protein; GO, Gene Ontology; HisRS, histidyl-tRNA synthetase; IQR, InterQuartile Region; TAP, Tandem Affinity Purification.
(PDF)

S3 Fig Interaction of HisRS with RNA by UV cross-linking. Strain expressing HisRS-TAP was subjected to UV illumination, to covalently cross-link protein–RNA interactions. RNA was then digested by three different concentrations of RNaseI (+++ [0.04 U/μl], ++ [0.008 U/μl], + [0.004 U/μl]). The TAP-tagged HisRS were purified together with the bound RNA fragments, and RNAs were radioactively labeled. Cross-linked HisRS–RNA complexes were resolved on SDS-PAGE and transferred to a membrane. A) Ponceau red protein staining of the membrane. Arrow indicates the band corresponding to HisRS-TAP, showing similar amounts of isolated protein in all samples. B) Autoradiography of the membrane, revealing bound HisRS–RNA complexes. An increase in HisRS apparent size is observed due to association with RNA. C) HisRS–RNA complexes of each sample were cut from the membrane, and RNA was recovered from the membrane by digesting the protein with proteinase K. RNA was separated using denaturing TBE Urea Polyacrylamide Gel and exposed to autoradiography. RNA species longer than 100 nts are clearly observed at the low RNaseI treatment. HisRS, histidyl-tRNA synthetase; TAP, Tandem Affinity Purification; TBE, Tris/Borate/EDTA.
(PDF)

S1 Table List of aaRS synthetases in *S. cerevisiae*, their intracellular localization, and whether they were detected in the indicated interactome studies. Notably, none of the exclusively mitochondrial aaRSs was detected in any of these studies. This is likely due to low polyadenylation of mitochondrial mRNAs, which is necessary for the RNA interactome isolation. Nevertheless, it confirms that mRNA association occurs while the cell is compartmentalized (i.e., before cellular lysis), hence no binding of mitochondrial aaRSs to cytosolic, polyadenylated transcripts occurs. aaRS, aminoacyl-tRNA synthetase.
(PDF)

S2 Table Raw RIP-seq data. Mapped unique reads for each GeneID in the S288c genome. The file includes several sheets. Sheet 1: uniquely mapped reads for all RIP-seq experiments. Sheet 2: raw read counts normalized to RPM. Sheet 3: HisRS background-corrected RIP efficiency. Transcripts with normalized read coverage <15 in the Input samples, and noncoding RNAs were filtered out. Bound sample were then divided by its corresponding Input sample to calculate RIP efficiency, and HisRS RIP efficiency was corrected to the Untagged RIP efficiency. Sheet 4: GlnRS background-corrected RIP efficiency, calculated as in Sheet 3. Sheet 5: ValRS background-corrected RIP efficiency, calculated as in sheet 3. Sheet 6: GluRS background-corrected RIP efficiency, calculated as in Sheet 3. Sheet 7: MetRS background-corrected RIP efficiency, calculated as in Sheet 3. Sheet 8: *arc1Δ* MetRS background-corrected RIP efficiency, calculated as in Sheet 3. GlnRS, glutamine-tRNA synthetase; HisRS, histidyl-tRNA synthetase; RIP, RNA immunoprecipitation; RPM, reads per million; ValRS, valyl-tRNA synthetase.
(XLSX)

S3 Table mRNAs that are bound by several aaRSs. mRNAs that appeared larger than 1.5 IQR region among all strains that were subjected to the TAP RIP protocol were selected and names and their gene ID are presented. Sheet 1 includes the genes that were used to generate the Venn diagram in S2A Fig, Sheet 2 corresponds to S2B Fig, and Sheet 3 corresponds to S2C Fig. aaRS, aminoacyl-tRNA synthetase; IQR, InterQuartile Region; RIP, RNA immunoprecipitation; TAP, Tandem Affinity Purification.
(XLSX)

S4 Table aaRS bound transcripts GO Term. Transcripts bound by a single aaRS or bound by two aaRSs were used to generate GO Term (using SGD GO Term Finder Version 0.86). aaRS, aminoacyl-tRNA synthetase; GO, Gene Ontology.
(DOCX)

S5 Table List of primers used in this work. (DOCX)

S6 Table Raw data for RT-qPCR assays, 35S methionine labeling, western and northern analysis, and polysome quantifications. Data are split into sheets according to the relevant figure. RT-qPCR, reverse transcription quantitative PCR.
(XLSX)

## Abbreviations

3AT: 3 aminotriazole
aaRS: aminoacyl tRNA synthetase
ABD: anticodon-binding domain
ADH1: alcohol dehydrogenase 1
AME: Arc1 methionyl glutamyl tRNA synthetase complex
AspRS: aspartyl-tRNA synthetase
CD: catalytic domain
CRISPR/Cas9: clustered regularly interspaced short palindromic repeats/ CRISPR-associated protein 9
IP: immunoprecipitation
FMRP: fragile X mental retardation 1
fRIP: fragmentation RIP
FT: flow-through
GFP: green fluorescent protein
GluRS: glutamyl-tRNA synthetase
GlnRS: glutamine-tRNA synthetase
GWIPS: genome-wide information on protein synthesis
HisRS: histidyl-tRNA synthetase
IQR: InterQuartile Range
LW: last washing
MetRS: methionyl-tRNA synthetase
mRBP: mRNA-binding protein
OD_254_: optical density
PAGE: polyacrylamide gel
RBP: RNA-binding protein
RIP: RNA immunoprecipitation
RT-qPCR: reverse transcription quantitative PCR
TAP: Tandem Affinity Purification
ThrRS: threonyl-tRNA synthetase
ValRS: valyl-tRNA synthetase
WT: wild-type
GluProRS: glutamyl-prolyl-tRNA synthetase
Cp mRNA: ceruloplasmin mRNA
GO: Gene Ontology
