# Supplementary figures and images for: mRNA association by aminoacyl tRNA synthetase occurs at a putative anticodon mimic and autoregulates translation in response to tRNA levels
Source: PLoS Biol. 2019 May 17;17(5):e3000274. doi: 10.1371/journal.pbio.3000274 (PMC6542539; doi:10.1371/journal.pbio.3000274)

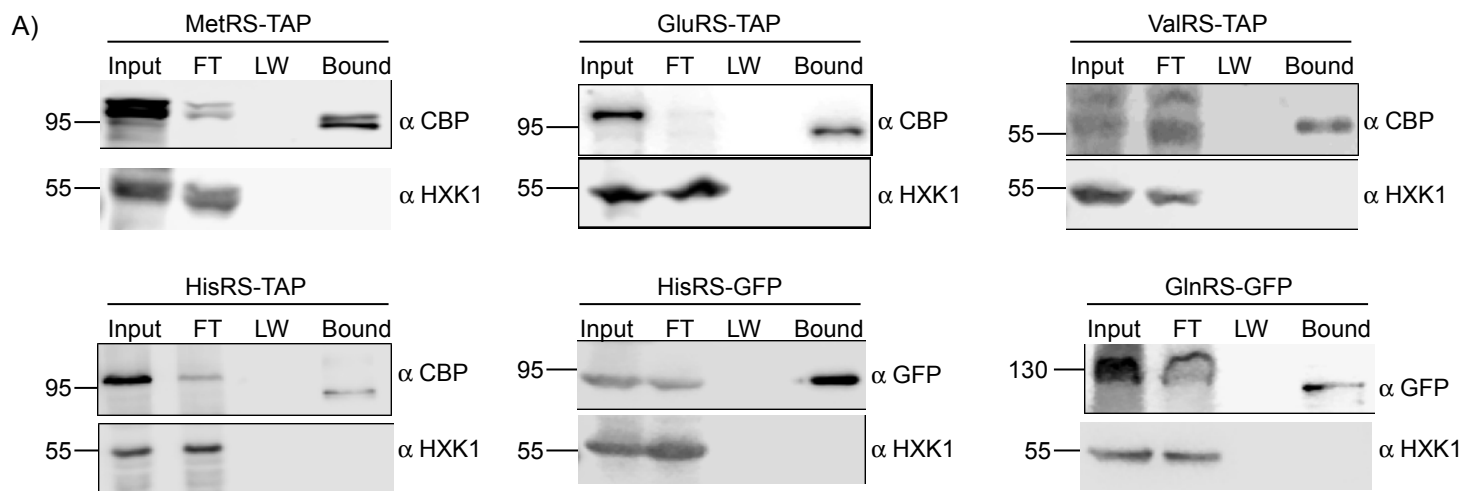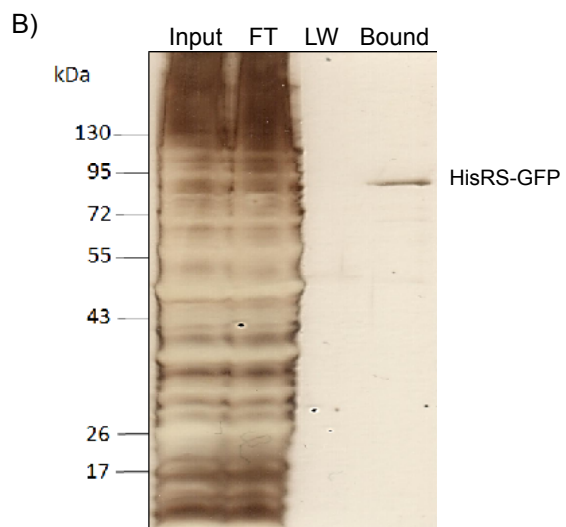

Supplement: S1 Fig — Yeast strains expressing tagged aaRSs were subjected to RIP using the TAP or GFP-Trap beads. Protein samples from the total cellular lysate (Input), unbound FT, LW step, and the eluted fraction (Bound) were resolved on PAGE followed by western analysis with the indicated antibodies (A) or stained with Silver stain (B). Equivalent amounts were loaded except of the Bound sample that was three times larger. Size markers are shown to the left. aaRS, aminoacyl-tRNA synthetase; FT, flow-through; GFP, green fluorescent protein; LW, last washing; RIP, RNA immunoprecipitation; TAP, Tandem Affinity Purification. (PDF) [file pbio.3000274.s001.pdf]

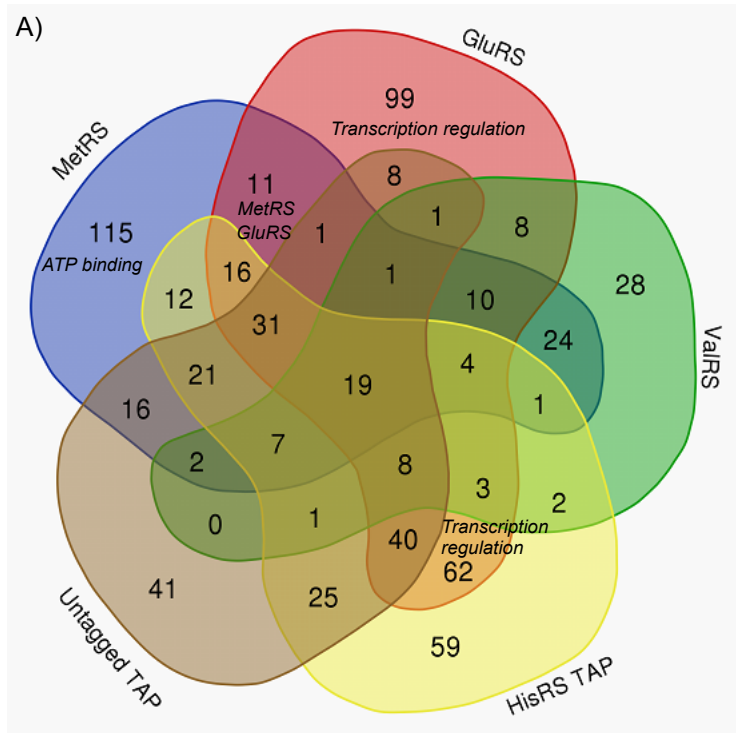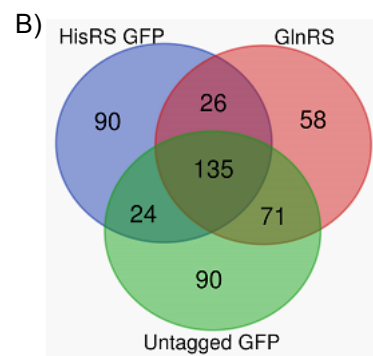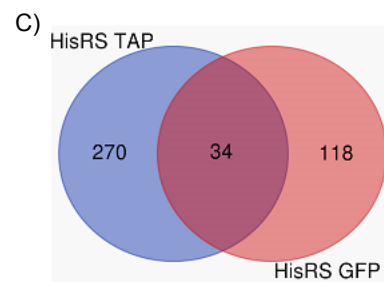

Supplement: S2 Fig — Venn diagrams were generated from the mRNAs that appeared within the 1.5 IQR region among all strains that were subjected to the TAP protocol (A), GFP-Trap protocol (B) and for HisRS tagged with either TAP of GFP (C). Shared genes or GO terms are indicated. aaRS, aminoacyl-tRNA synthetase; GFP, green fluorescent protein; GO, Gene Ontology; HisRS, histidyl-tRNA synthetase; IQR, InterQuartile Region; TAP, Tandem Affinity Purification. (PDF) [file pbio.3000274.s002.pdf]

A)

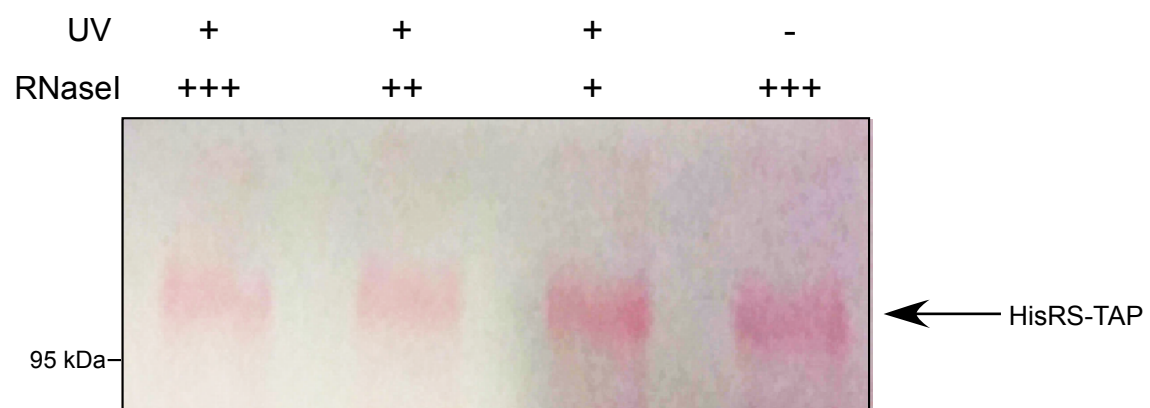

B)

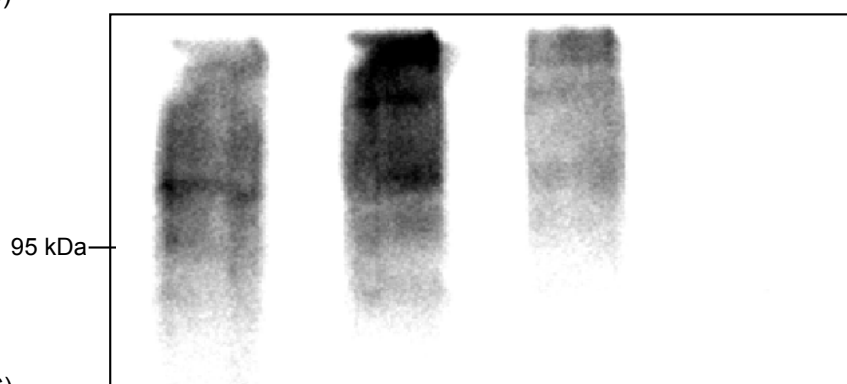

C)

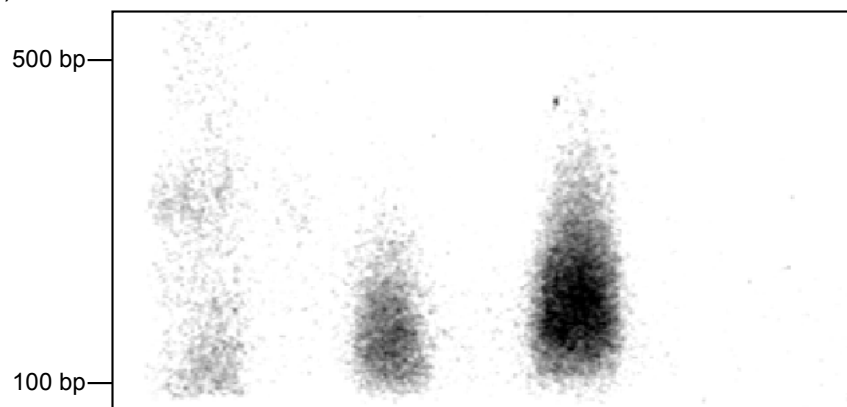

Supplement: S3 Fig — Strain expressing HisRS-TAP was subjected to UV illumination, to covalently cross-link protein–RNA interactions. RNA was then digested by three different concentrations of RNaseI (+++ [0.04 U/μl], ++ [0.008 U/μl], + [0.004 U/μl]). The TAP-tagged HisRS were purified together with the bound RNA fragments, and RNAs were radioactively labeled. Cross-linked HisRS–RNA complexes were resolved on SDS-PAGE and transferred to a membrane. A) Ponceau red protein staining of the membrane. Arrow indicates the band corresponding to HisRS-TAP, showing similar amounts of isolated protein in all samples. B) Autoradiography of the membrane, revealing bound HisRS–RNA complexes. An increase in HisRS apparent size is observed due to association with RNA. C) HisRS–RNA complexes of each sample were cut from the membrane, and RNA was recovered from the membrane by digesting the protein with proteinase K. RNA was separated using denaturing TBE Urea Polyacrylamide Gel and exposed to autoradiography. RNA species longer than 100 nts are clearly observed at the low RNaseI treatment. HisRS, histidyl-tRNA synthetase; TAP, Tandem Affinity Purification; TBE, Tris/Borate/EDTA. (PDF) [file pbio.3000274.s003.pdf]
