# Supplementary material for: mRNA association by aminoacyl tRNA synthetase occurs at a putative anticodon mimic and autoregulates translation in response to tRNA levels
Source: PLoS Biol. 2019 May 17;17(5):e3000274. doi: 10.1371/journal.pbio.3000274 (PMC6542539; doi:10.1371/journal.pbio.3000274)
Supplement: S1 Table — Notably, none of the exclusively mitochondrial aaRSs was detected in any of these studies. This is likely due to low polyadenylation of mitochondrial mRNAs, which is necessary for the RNA interactome isolation. Nevertheless, it confirms that mRNA association occurs while the cell is compartmentalized (i.e., before cellular lysis), hence no binding of mitochondrial aaRSs to cytosolic, polyadenylated transcripts occurs. aaRS, aminoacyl-tRNA synthetase. (PDF) [file pbio.3000274.s004.pdf]

Gene	Amino Acid	local	Mitchell et al [11]	Beckmann et al [7]	Matia-González et al [10]
**HTS1 / YPR033C**	His	Cyt+Mit	V	V	V
**GUS1 / YGL245W**	Glu	Cyt	V	V	V
**GLN4 / YOR168W**	Gln	Cyt+Mit	V	V	V
**TYS1 / YGR185C**	Tyr	Cyt	V	V	V
**KRS1 / YDR037W**	Lys	Cyt	V	V	V
**SES1 / YDR023W**	Ser	Cyt	-	V	V
**MES1 / YGR264C**	Met	Cyt	-	V	V
**THS1 / YIL078W**	Thr	Cyt	-	V	V
**VAS1 / YGR094W**	Val	Cyt+Mit	-	V	V
**YHR020W**	Pro	Cyt	-	V	V
**CDC60 / YPL160W**	Leu	Cyt	-	V	V
**DED81 / YHR019C**	Asn	Cyt	-	V	V
**DPS1 / YLL018C**	Asp	Cyt	-	V	V
**YDR341C**	Arg	Cyt	-	V	V
**ILS1/ YBL076c**	Ile	Cyt	-	V	V
**ALA1 / YOR335C**	Ala	Cyt+Mit	-	V	V
**FRS2 / YFL022C**	Phe	Cyt	-	V	V
**FRS1 / YLR060W**	Phe	Cyt	-	-	V
**WRS1 / YOL097C**	Trp	Cyt	-	-	V
**GRS1 / YBR121C**	Gly	Cyt+Mit	-	-	V
**YNL247W**	Cys	Cyt	-	V	-
**SLM5 / YCR024C**	Asn	Mit	-	-	-
**MST1 / YKL194C**	Thr	Mit	-	-	-
**MSR1 / YHR091C**	Arg	Mit	-	-	-
**ISM1 / YPL040C**	Ile	Mit	-	-	-
**MSF1 / YPR047W**	Phe	Mit	-	-	-
**NAM2 / YLR382C**	Leu	Mit	-	-	-
**MSE1 / YOL033W**	Glu	Mit	-	-	-
**MSK1 / YNL073W**	Lys	Mit	-	-	-
**MSY1 / YPL097W**	Tyr	Mit	-	-	-
**MSW1 / YDR268W**	Trp	Mit	-	-	-
**MSM1 / YGR171C**	Met	Mit	-	-	-
