# Supplementary material for: mRNA association by aminoacyl tRNA synthetase occurs at a putative anticodon mimic and autoregulates translation in response to tRNA levels
Source: PLoS Biol. 2019 May 17;17(5):e3000274. doi: 10.1371/journal.pbio.3000274 (PMC6542539; doi:10.1371/journal.pbio.3000274)
Supplement: S4 Table — Transcripts bound by a single aaRS or bound by two aaRSs were used to generate GO Term (using SGD GO Term Finder Version 0.86). aaRS, aminoacyl-tRNA synthetase; GO, Gene Ontology. (DOCX) [file pbio.3000274.s007.docx]

| **Corrected P-value**  (Top 5) | **Gene Ontology Term**  (Function) | **Bound transcripts**  (1.5 times IQR) | **aaRS(s)** |
| --- | --- | --- | --- |
| 4.07e-13 | Binding (GO:0005488) | YJL080C YOL098C YJL200C YGR271W YFL004W YHR098C YGR240C YJR140C YGR224W YML072C YMR217W YMR080C YAL036C YOR108W YEL032W YBR038W YDR135C YNR016C YMR006C YOR151C YJL050W YDR119W YBR208C YDR234W YLR384C YDL063C YLL034C YHR074W YGL234W YHR186C YDR385W YDR101C YKL210W YOR335C YGR159C YDL227C YHR023W YEL055C YML063W YAL026C YBL008W YGR098C YBL017C YMR266W YIL125W YOR270C YGR279C YDR170C YMR129W YGR184C YOR116C YOR291W YPL006W YPL231W YJR143C YNL262W YKL004W YGL195W YOR086C YHR128W YDR127W YPR010C YEL022W YOR133W YBR263W YDR212W YAL029C YLR305C YNL132W YCR057C YBR084W YGL173C YCR032W YFL007W YDR180W YPL217C YER105C YDL112W YHR041C YBR115C YHR047C YGL201C YJR132W YKL014C YNL102W YJL039C YOR229W YKL073W YHR042W YMR189W YOR048C YBR275C YNL085W YJL109C YNL287W YJR131W YLL048C YOL069W YMR207C YDR238C YMR229C YMR308C YKL176C YOR341W YBR218C YML056C YGL026C YGL022W YPL093W YOR011W YJR064W YLR450W YGR157W YHR020W YGR032W | MetRS-TAP |
| 8.03e-13 | Small molecule binding (GO:0036094) |  |  |
| 2.21e-11 | Ion binding (GO:0043167) |  |  |
| 4.09e-10 | Carbohydrate derivative binding  (GO:0097367) |  |  |
| 5.73e-10 | Nucleotide binding (GO:0000166) |  |  |
| 3.27e-05 | Transcription regulator activity  (GO:0140110) | YGL021W YOR195W YGR097W YOR178C YIL135C YBR086C YHR080C YLR206W YKL204W YMR273C YMR043W YFR019W YCR033W YML020W YNL186W YGL071W YBL085W YAR042W YDR217C YMR164C YDR270W YER033C YHR079C YDR359C YBR108W YJR151C YMR279C YIR010W YMR016C YKL043W YJL127C YDL048C YHR143W YOR191W YER129W YML032C YGL023C YML076C YOR156C YDR326C YLR337C YER008C YLL010C YEL043W YMR172W YLR116W YDR464W YBL023C YML081W YHR161C YPR115W YJL019W YBR065C YGL178W YBR225W YOR267C YLR219W YIL149C YOR181W YOR017W YIR019C YER158C YHR177W YNL298W YLR096W YOR066W YCL061C YNL124W YDR409W YKR103W YPL016W YKL105C YLR278C YMR216C YOR329C YOR217W YPR095C YJL129C YGR249W YER169W YCR089W YAL031C YBR028C YLR055C YLR223C YER047C YNL187W YDR207C YNL103W YLR371W YDL056W YLL021W YIL031W YDL223C YNL321W YKL050C YKL068W YJR059W YML117W | GluRS-TAP |
| 0.00019 | Sequence-specific DNA binding (GO:0043565) |  |  |
| 0.00036 | DNA binding transcription factor activity (GO:0003700) |  |  |
| 0.00136 | DNA binding (GO:0003677) |  |  |
| 0.00147 | Lipid binding (GO:0008289) |  |  |
| **-** | **-** | YPR116W YDR213W YOR060C YBR019C YJL083W YKR031C YKL038W ICR1 YBL074C YGR068C YFL055W YLR398C YNL139C YDR216W YMR280C YNR069C YKL203C YER172C YJR129C YNL278W YJL005W YLR422W YDR334W YPL167C YMR053C YKL197C YDL238C YDR314C | ValRS-TAP |
| **-** | **-** | YEL064C YER060W-A YGR204W YDL149W YGR097W YER013W YHR105W YOL155C YJL019W YGL178W YPR044C YOR336W YEL074W YOR301W YGR126W YLR245C YDR097C YJR041C YKR045C YDL109C YOL051W YAR042W YBL014C YDR505C YBR117C YER164W YOR137C YIL150C YDR274C YGR188C YLR357W YER033C YJR131W YDR359C YLR004C YCR068W YBR059C YFL033C YJL127C YPR119W YJL105W YKL071W YMR054W YML109W YOR156C YNL187W YLR190W YDR093W YGL035C YDR247W YOR291W YNL014W YPL128C YER167W YNL321W YOL011W YGL064C YLR291C | GlnRS-GFP |
| 0.00155 | Lipid kinase activity (GO:0001727) | YPL194W YPR010C YLL038C YCR061W YDR186C YER070W YOR171C YNL304W YHR056C YNL106C YOR334W YBR054W YKR102W YGL094C YBL101C YCR100C YBR117C YDL220C YLL019C YFL031W YLR313C YLR044C YCR068W YAL048C YPR033C YML111W YDL160C YPR032W YIL107C YDL140C YJL181W YAL038W YPL172C YLR260W | HisRS TAP  &  HisRS -GFP |
| 0.00155 | Sphinganine kinase activity (GO:0008481) |  |  |
| 0.00155 | D-erythro-sphingosine kinase activity (GO:0017050) |  |  |
| 0.00127 | Ion binding (GO:0043167) | YBR097W YIL009W YDL066W YPR145W YGL167C YNL106C YCR106W YMR079W YBL037W YFL031W YNL085W YMR304W YOL100W YOL076W YAL048C YGR098C YGR054W YGL197W YOR011W YKL129C YPR032W YMR300C YLR024C YGL131C YLR116W YOR168W | HisRS  &  GlnRS (GFP) |
| 0.00851 | Binding (GO:0005488) |  |  |
| 4.52e-06 | Transcription regulatory region DNA binding (GO:0044212) | YDR389W YER060W-A YGR162W YKR090W YPL089C YKL032C YGR023W YOL019W YDR186C YGL014W YHR206W YER123W YDL224C YER151C YLR019W YLR067C YCR084C YOL100W YLR006C YHL029C YAL048C YCL044C YDR515W YHL008C YLL051C YOR372C YLR139C YOR247W YHR084W YML010W YOR140W YJL168C YHR205W YPR104C YNL068C YPR065W YDR293C YCL063W YDL109C YKR102W YLR131C YGL180W YER111C YMR047C YML027W YLR313C YJL078C YLR399C YHR158C YBR059C YNR008W YCR077C YKR092C YDR143C YER040W YAL021C YMR054W YOL036W YKR050W YNL197C YDR077W YJL181W | GluRS + HisRS (TAP) |
| 5.19e-06 | Regulatory region nucleic acid binding (GO:0001067) |  |  |
| 9.25e-06 | Transcription regulatory region sequence-specific DNA binding (GO:0000976) |  |  |
| 6.17e-05 | Transcription regulator activity (GO:0140110) |  |  |
| 0.00023 | RNA polymerase II regulatory region sequence-specific DNA binding (GO:0000977) |  |  |
| **-** | **-** | YOR109W YGL140C YGR126W YFL052W YDR457W YGR094W YOR371C YCR093W YLR024C YBL079W YBR295W YGR281W YKL182W YBR136W YOR336W YLR454W YDR274C YBL004W YGR061C YBR140C YPL082C YHL030W YLL040C YKL215C | MetRS & ValRS |
