# Supplementary material for: mRNA association by aminoacyl tRNA synthetase occurs at a putative anticodon mimic and autoregulates translation in response to tRNA levels
Source: PLoS Biol. 2019 May 17;17(5):e3000274. doi: 10.1371/journal.pbio.3000274 (PMC6542539; doi:10.1371/journal.pbio.3000274)
Supplement: S5 Table — (DOCX) [file pbio.3000274.s008.docx]

**Supplementary Table 5: List of primers used in this work**

| **Semi- quantitative PCR primers** | |
| --- | --- |
| **Primer Name** | **Primer sequence** |
| HisRS F | GTACGAAGGATTGGGCTGATAG |
| HisRS R | GTGGAGCAGATGCAGAAGTAA |
| Actin F | ACGTTACCCAATTGAACACGGTAT |
| Actin R | TTAGAAACACTTGTGGTGAACGAT |
| MetRS F | GAAGAAACATCCTGCCCATTTG |
| MetRS R | TTCCGTGGCAGTACCATATTC |
| GluRS F | AGGTGTTACCCACGCTTTAC |
| GluRS R | CCAATCTGTAGTAGCCCTTTCTT |
| ValRS F | GAAGGCGAAGAACACGATAGAA |
| ValRS R | CAGTGGCAGGTGGTTGATAA |
| **qPCR primers** | |
| **Primer Name** | **Primer sequence** |
| HisRS F | GCCATGACTAAGGGTCGTATG |
| HisRS R | ACTGGTCAAACCTTCCACTAAA |
| Actin F | GATTCTGAGGTTGCTGCTTTG |
| Actin R | ACCGACGATAGATGGGAAGA |
| MetRS F | GCCTAAGCCAAACGAAAGAAAC |
| MetRS R | GCTGAAAGAACACTACCGATGA |
| **MS2 Loops tagging primers** | |
| HisRS MS2L F | GATGGTCTAAATGAGGTCACTCGTTTAATTAAAGGATTAC  ATCATCACCATCACCACTAACGGGCCCTATATATGGATCC |
| HisRS MS2L R | GTTTTACGAAGGTTTGTAAATACATGAGCTATCAAGTTTA  GCGCGCAGATCTAATGAACC |
